# Avian Candidiasis: A Comprehensive Review of Pathogenesis, Diagnosis, and Control

**DOI:** 10.3390/vetsci13020171

**Published:** 2026-02-09

**Authors:** Michelyne Haroun, Christophe Tratrat, Roshmon Thomas Mathew, Muhammad Munir, Muhammad Naeem Sattar, Mohamed Shawky, Hafedh Kochkar, Ouda Nasser Aldakhilallah, Abdul Ghafoor, Khalid G. Biro Turk, Athina Geronikaki, Hesham S. Ghazzawy

**Affiliations:** 1 Fish Resources Research Center, King Faisal University, Al-Ahsa 31982, Saudi Arabia; rmathew@kfu.edu.sa (R.T.M.); oaldakhilalla@kfu.edu.sa (O.N.A.); 2 Department of Pharmaceutical Sciences, College of Clinical Pharmacy, King Faisal University, Al-Ahsa 31982, Saudi Arabia; ctratrat@kfu.edu.sa; 3 Date Palm Research Center of Excellence, King Faisal University, Al-Ahsa 31982, Saudi Arabia; 4 Central Laboratories, King Faisal University, Al-Ahsa 31982, Saudi Arabia; mnsattar@kfu.edu.sa; 5 Avian Research Center, King Faisal University, Al-Ahsa 31982, Saudi Arabia; mabdelmoaty@kfu.edu.sa; 6 Basic & Applied Scientific Research Center, Imam Abdulrahman Bin Faisal University, Dammam 31441, Saudi Arabia; hbkochkar@iau.edu.sa; 7 Water and Environmental Studies Center, King Faisal University, Al-Ahsa 31982, Saudi Arabia; aghafoor@kfu.edu.sa (A.G.); kturk@kfu.edu.sa (K.G.B.T.); 8 Department of Pharmaceutical Chemistry, School of Pharmacy, Aristotle University of Thessaloniki, 54124 Thessaloniki, Greece; geronik@pharm.auth.gr

**Keywords:** candidiasis, budding, pseudohypha, hypha, nystatin, pathogenesis, morphogenesis, invasion

## Abstract

This review is a comprehensive investigation of avian candidiasis, mainly caused by *Candida albicans*, although the prevalence of non-albicans *Candida* species has increased in domestic and wild birds. Avian candidiasis causes significant economic losses in poultry production through increased mortality, cost of treatments, and reduced growth rates, particularly in young birds and intensive farming operations. The pathogenesis section provides a description of the molecular virulence factors such as adhesin-mediated attachment (ALS, Agglutinin-Like Sequence family; HWP1, Hyphal Wall Protein 1), yeast-to-hypha morphogenesis, tissue damage by Candidalysin, biofilm formation on mucosal and abiotic surfaces, and secreted hydrolytic enzymes including secreted aspartyl proteinases (SAPs) and phospholipases. The identified predisposing factors include immunosuppression, malnutrition, abuse of antibiotics, bad husbandry, and crop stasis. The diagnostic methods discussed encompass cytological analysis and fungal culture on selective media to more sophisticated methods of molecular analysis (PCR, MALDI-TOF MS, and NGS). Antifungal susceptibility investigations indicate that nystatin and amphotericin B are still very effective against most avian isolates and that resistance to the azoles is on the rise, especially with respect to the non-albicans *Candida* species. Nystatin is still the first-line treatment of localized infections; azoles are still used for resistant or systemic infections despite their hepatotoxicity. Sanitation, proper nutrition, and proper use of antimicrobials are essential to prevent diseases. The knowledge gaps comprise the absence of avian-specific pharmacokinetic information, poor knowledge of species-species virulence phenotypes, and the lack of point-of-care diagnostics. The need to have integrated One Health surveillance systems is emphasized by the zoonotic potential of the avian *Candida* reservoirs.

## Simple Summary

Candidiasis is a fungal infection that is caused by *Candida* yeasts, mostly *Candida* albicans, and affects domestic and wild birds worldwide. The disease tends to affect young chicks, stressed birds, and birds with weakened immune systems, especially under poor hygiene conditions, excessive antibiotic use, or nutritional deficiencies. Although of clinical significance, no single volume was accessible to summarize existing knowledge on this avian disease. This review fills this gap by studying the different *Candida* species identified in birds and the predisposing factors. Molecular pathways involving the fungus’s tissue binding, switch from yeast to invasive hyphae, toxin release, and formation of drug-resistant communities are investigated. Clinical symptoms comprising white spots in the mouth and distension of the crop to severe systemic disease are detailed, as well as diagnostic approaches, starting with simple microscopy and ending with advanced molecular methods. Treatment options are considered, though nystatin is still the choice of localized infections, whereas prevention by means of proper hygiene, proper nutrition, and responsible use of antibiotics is still central. Since birds have the potential to spread these yeasts to humans, this review highlights the importance of integrated surveillance, which is beneficial to veterinarians, poultry producers, wildlife managers, and public health professionals.

## 1. Introduction

Avian candidiasis is an opportunistic fungal infection primarily caused by the yeasts of the *Candida* genus, mostly *Candida albicans* (*C. albicans*) [1,2]. The disease, also known as thrush, sour crop, or crop mycosis, occurs sporadically worldwide [3]. It has been observed in a large variety of avian birds, such as domestic poultry (chickens, turkeys, geese, ducks, and quails), companion birds (parrots, cockatiels, and budgerigars), and wild birds (pigeons and birds of prey) [4]. Infection mostly occurs in the upper gastrointestinal tract (oropharynx, esophagus, and crop), but in severe cases, it may implicate the respiratory system, central nervous system, and skin as well [5]. Candidiasis may present either as a primary infection or secondary infection together with other underlying conditions [5]. Birds that are less than three weeks old are highly vulnerable, and infected poultry can exhibit retarded development, depression, diarrhea, and dehydration [3]. Morbidity and mortality rates are relatively low, but the disease may lead to economic losses owing to low growth rates and the cost of treatments, particularly in flocks having immunocompromised conditions [3,6]. Lack of effective vaccines and the emergence of antifungal resistance have made the control of avian candidiasis a challenging task, and hence, prevention of this issue by means of proper husbandry and biosecurity is crucial [3,4]. As feral pigeons deposit their droppings anywhere and nest in unlikely places such as air conditioning systems, trees, and cornices, these locations could represent points of dissemination to hospitals, schools, or other high-risk settings [7]. Thus, pigeons and their droppings act as carriers and reservoirs of *Candida* spp. and other zoonotic yeasts and may be a source of infection, specifically with respect to immunocompromised hosts [8,9]. The *Candida* genus includes ascomycetous yeasts and is being classified along phylogenetic lines [10]. Members of this group of yeasts lack unique morphological traits that differentiate them from other asexually or sexually reproducing ascomycetous yeast genera [11]. They reproduce asexually through budding and may form pseudohyphae or true hyphae [12], but these are typical forms, among other ascomycetous yeasts, appearing as non-distinctive to classification [13]. In its classical sense, it is a member of the *Saccharomycotina* family comprising over 400 species [10], of which 15 were recognized as infections in humans and animals [14,15] and occurring in almost all families of the subclass [10]. Some *Candida* species (spp.) present a major significance in avian medicine as prominent pathogens comprehending *C. tropicalis*, *C. krusei*, *C. glabrata*, and *C. parapsilosis* [8]. They are additionally commonly perceived as comprised in the normal flora made up of healthy birds [5] and are thus regarded as common commensal fungus that colonizes the oropharynx, gastrointestinal tract, and the skin of avian species. From local and systemic to environmental and hereditary, diverse causes lead to disorders in the normal homeostasis of *Candida*, inducing a conversion from normal microbiota to pathogenic disorders [5]. The pathophysiology evolution of the onset and infection progression is also impacted by *Candida*’s virulence traits that promote the development of candidiasis [8]. This review seeks to give an in-depth description of avian candidiasis, comprising etiology, pathogenesis, predisposing factors, clinical manifestations, diagnostic, treatment, and preventive approaches.

## 2. Pathogenesis

### 2.1. Candida Species

Fungal pathogens incur an estimated 6.5 million invasive infections and about 3.8 million deaths annually worldwide, with *Candida* species alone contributing about 1.6 million bloodstream infections and close to 1 million deaths annually [16]. *Candida* species are globally presently known as the cause of most human and animal fungal infections [17]. The *Candida* genus is still extremely phylogenetically divergent, as it comprises a wide range of phylogenetically unrelated anamorphic fungi [18]. Most *Candida* species are non-pathogenic and widely distributed in nature, e.g., in water and soil, or associated with food, plants, insects, or animals [11,19]. These species are also frequently found as common saprophytic constituents of the normal human and animal microflora of healthy humans and animals and are thus considered commensal and facultatively pathogenic [20,21]. Birds are particularly susceptible to gastrointestinal candidiasis, which has been observed in geese, ducks, broiler chickens, guinea fowls, pheasants, quails, pigeons, parrots, and birds of prey [8,22,23,24,25,26,27,28]. In healthy avian hosts, non-albicans *Candida* species (NACS) are being increasingly identified as causes of infection. They include *C. lusitaniae*, *C. lambica*, *C. glabrata*, *C. tropicalis*, *C. stellatoidea*, *C. parapsilosis*, *C. rugosa,* and *C. krusei*, isolated in both symptomatic and asymptomatic birds [23,29,30]. These yeasts are common in the bird gut’s microbiota and can cause gastrointestinal issues like diarrhea or regurgitation, especially if the gut’s normal environment is disrupted [23]. Various domestic animals (e.g., cattle, horses, pigs, cats, dogs, avian species) are susceptible hosts to *Candida* infections, raising questions about their role in the transmission of these yeasts to humans, especially immunocompromised individuals [31]. Table 1 displays some examples of isolated *Candida* spp. and their avian hosts.

### 2.2. Predisposing Factors of Avian Species to Candidiasis

In birds, candidiasis predominantly manifests as an alimentary tract disease with the fungus colonizing both the skin, mucosal surfaces, and digestive tract of healthy avian hosts as a commensal organism [41]. Under normal physiological conditions, *C. albicans* develops a harmonious mix of energy and metabolites with the host microbiota and dwells in the upper part of the gastrointestinal tract as a commensal [42,43,44]. This balance is, however, destroyed when the immune defenses of the bird are threatened by stress, malnourishment, or poor husbandry, allowing *Candida* to develop into a pathogenic stage [5,42,45,46,47]. Therefore, the illness develops due to weakening of the local or systemic defenses, which permits the growth and invasion of the mucosa, which is classically found in the oral cavity (thrush), crop (crop mycosis), and proximal esophagus [48]. Core conditions leading to a change in commensalism–pathogenicity balance include the extended or inappropriate administration of antibacterial therapy to disrupt the bacterial flora, high-carbohydrate/spoiled feed, lack of good hygiene, crop stasis (e.g., after hand-rearing in psittacines and passerines or gavage feeding in waterfowl), and protein and vitamin deficiencies, in addition to immunosuppression. These conditions enable a quick increase in yeast cells, biofilms, and mucosal destruction [49]. The combination of these conditions provides the circumstances in which conventionally commensal *Candida* spp. may become pathogenic and cause clinical candidiasis [19,50,51].

#### 2.2.1. Environment, Age, Immune Status, and Stress

Concomitant diseases and chronic stress can compromise avian immune function through multiple pathways. The immune response in birds can be modulated by both intrinsic factors (such as sex and age) and extrinsic factors (such as exposure to toxicants and climatic conditions), hence affecting their vulnerability to infectious challenges and the effectiveness of vaccinations [52,53,54]. Examples of toxicants that are known to induce avian immunosuppression include heavy metals (lead, cadmium, and arsenic) [55,56,57], mycotoxins (aflatoxins and fumonisins) [58], and pesticides (organophosphates) [59]. With respect to age, young poultry chicks and unweaned hand-reared psittacine and passerine chicks and neonates are likely to be the most affected because their immune systems are underdeveloped [60,61,62]. The stressors that predispose birds synergistically include pollution [63], loss of habitats [63,64], transport [65], climate change [66], and parasitism [67,68,69]. Additionally, the use of immunosuppressive drugs can weaken the immune system, making birds more susceptible to opportunistic infections by fungi [70,71]. This vulnerability is further enhanced by environmental stress factors, i.e., heat, congestion, and poor ventilation, which trigger physiological stress responses that hinder avian immunity [72,73,74]. On top of this, the season may contribute to it, with some studies showing a drop in *Candida* incidence in spring, which may be due to the fact that at this time of the year, birds may be more immunocompetent [50,75,76].

#### 2.2.2. Malnutrition

Vitamin A plays a crucial role in preserving the integrity of epithelial and mucosal barriers that form the first line of defense against pathogens [77]. Lack of this micronutrient leads to squamous metaplasia and keratinization of the mucosal surfaces of the upper digestive tract, hence disrupting the protective barrier and permitting opportunistic fungi like *Candida* to colonize and invade host tissues [3,5].

Trace minerals are also critical for host defense against fungal pathogens. Zinc plays a crucial role in the normal functioning of heterophils, mononuclear phagocytes, and T lymphocytes of poultry. Zinc insufficiency impairs phagocytic activities and T cell maturation, thereby leaving poultry vulnerable to infections [78]. Selenium is also needed for the antioxidant selenoenzymes, including glutathione peroxidase, which protect phagocytes during the oxidative burst; selenium deficiency impedes the phagocytic potential of macrophages and heterophils’ ability to kill ingested pathogens [79,80]. Consequently, a lack of these trace minerals compromises the innate immunity of the avian organisms and predisposes them to opportunistic fungi such as candidiasis [3,5].

#### 2.2.3. Medical Therapy

The broad-spectrum use of antibiotics can decimate the normal beneficial gut/crop microflora, creating ecological niches that foster prompt yeast proliferation [81,82]. Alteration or displacement of indigenous microbiota after immunosuppressive therapy or extreme surgery also enhances susceptibility in animals, imitating processes that are viewed in human candidiasis [83]. Likewise, indwelling catheters and total parenteral nutrition are identified as risk factors of fungal infections in different species, including birds, because of their severe effects on microbial ecology and host defenses. It is noteworthy that *C. parapsilosis* is often associated with secondary fungal infections of indwelling medical devices, which are frequent in birds (particularly companion birds such as psittacines) that receive treatments or fluid therapy [84,85].

#### 2.2.4. Husbandry Conditions

Poor nutrition habits, which include the use of contaminated utensils, syringes, or hand feeding (particularly in hand-reared psittacines and passerines), also cause oral inoculation with high yeast loads. The hygiene of drinkers and feeding stations is also a contributory factor. In addition, for birds, poor quality feed, unsanitary practices, and contaminated water sources open the door to pathogens and can compromise their biological defense of living environments [86,87]. In particular, parasitic infestations, unhygienic or moldy feed, and unfavorable living conditions pose a high risk of candidiasis, especially in young poultry chicks and hand-reared psittacine birds [88,89]. As an illustration, malnutrition, poor hygiene, and genetic predispositions are known to play a significant role in the development of fungal diseases in avian species [90]. In short, candidiasis in avian species is frequently a result of a dysbiosis, in which the development of *Candida* is facilitated by the lack of an innate defense in the host or environmental stability [49,60,91].

#### 2.2.5. Physiological Conditions

Normally, the crop should empty in 4–6 h in chicks [92]; delayed emptying or crop stasis results in a nutrient-abundant, warm habitat, which favors the growth of yeasts. Retained feed in the crop increases the risk of *Candida* 1 overgrowth [93,94]. This condition is extremely common in immune-compromised or young birds across all avian species, particularly poultry chicks and hand-reared psittacine nestlings, and can engender fungal or bacterial infections, fermentation, and digestive blockages [60]. This allows feed to stagnate in the crop longer than normal, thus paving the way to the increased risk of *Candida* spp. overgrowth, particularly *Candida albicans*, which thrives under such conditions and may induce crop thickening and emergence of white plaques in the oral cavity and subsequent infection [6,60].

### 2.3. Molecular Pathogenic Mechanisms

Originally, *C. albicans* is typically an innocent resident (a commensal) in the avian gastrointestinal tract [60]. However, it may be turned into a disease-causing agent (a pathogen) in case the host defenses are compromised, which can occur in immunocompromised birds [60]. A complex, integrated circuit of molecular systems orchestrates the pathogenicity of *Candida* in adhesion, morphogenesis, metabolic remodeling, enzyme secretion, and immune evasion. In fact, *Candida albicans* pathogenesis by definition represents a complex of adhesin networks, secreted effectors, morphogenetic switches, metabolic adaptation systems, and transcriptional regulators that, as a group, allow adhesion, tissue invasion, immune modulation, and persistence at mucosal locations, including the avian crop [95]. These systems play a central role in the avian crop environment for the transformation of a benign colonizer to a destructive pathogen switchover. *Candida* pathogenicity in avian hosts involves a complex of coordinated virulence factors, including surface adhesion and biofilm formation, yeast to hypha morphogenesis with Candidalysin-mediated tissue damage, and secreted hydrolases and membrane-active enzymes that degrade host defenses and regulate immunity through an interconnected system of epidemiological signaling pathways with the essentially mitogen-activated protein kinase (MAPK) and cyclic AMP-dependent protein kinase A (cAMP–PKA) cascades in addition to the transcriptional regulators that incorporate environmental cues: EFG1 (enhanced filamentous growth protein 1) and BCR1 (biofilm and cell wall regulator 1) [5,48,96,97].

#### 2.3.1. Adhesion and Early Colonization

**Figure 1:**
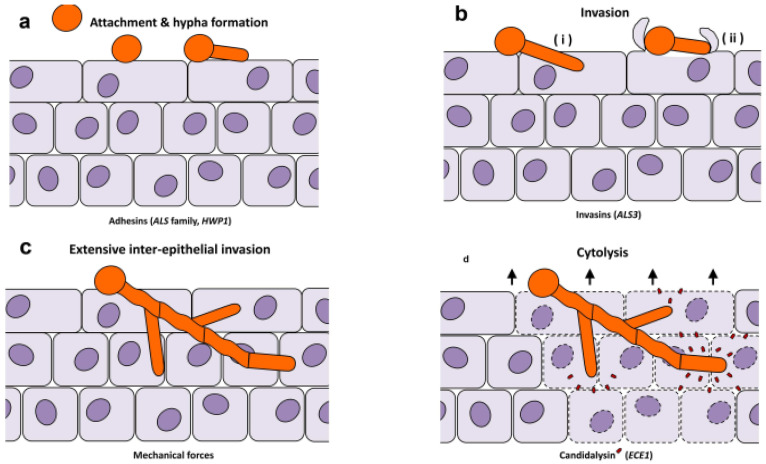
Sequential infection of *Candida albicans* of the epithelium. Attachment and hypha formation (**a**) facilitated by adhesin (ALS family, HWP1). (**b**) Invasion under 2 mechanisms: (**i**) active penetration via fungi and (**ii**) induced endocytosis via fungi and use of invasin (ALS3). Large-scale interepithelial invasion is encouraged by mechanical forces. (**c**) Extensive inter-epithelial invasion driven by mechanical forces. (**d**) Cytolysis and cell damage by *Candida albicans* Candidalysin (ECE1). Adapted from Wilson et al., PLoS Pathogens, 2016, licensed under CC BY 4.0 [98].

Early tissue invasion by *C. albicans* occurs in several sequential steps: adhesion to the epithelium by fungal adhesins [including Agglutinin-like sequence (ALS)], epithelial penetration and invasion by hyphae through active penetration and induced endocytosis, extensive inter-epithelial invasion propelled by mechanical forces, and epithelial cytolysis mediated by Candidalysin (detailed in Section 2.3.2) 111 (Figure 1a–d) [98]. Epithelial breach is then succeeded by vascular dissemination involving hyphal penetration of blood vessels and seeding of yeast cells into the bloodstream, eventually resulting in endothelial colonization and infiltration of the disease during dissemination in the blood [99].

The initial colonization is dependent on *Candida* cell wall fungal adhesins. *Candida* can express a range of significant adhesins, the most remarkable of which are the ALS family proteins (ALS1-ALS9) and hypha wall protein 1 (HWP1), which cause strong, species- and niche-specific binding to host epithelial ligands that comprise fibronectin and to epithelial cadherins that enable strong adherence to crop and esophageal mucosa, along with abiotic surfaces (feeding tubes and syringes used in psittacine and passerine hand-rearing). Interestingly, recent studies have disclosed the so-called enlarged and highly adhesive morphotype *Goliath* cells, variant cells of *C. albicans*, that exhibit a notable hyper-adherence to both biotic and abiotic surfaces and might promote early biofilm nucleation [100,101].

#### 2.3.2. Generation of Morphogenesis, Candidalysin, and Epithelial Injury

**Figure 2:**
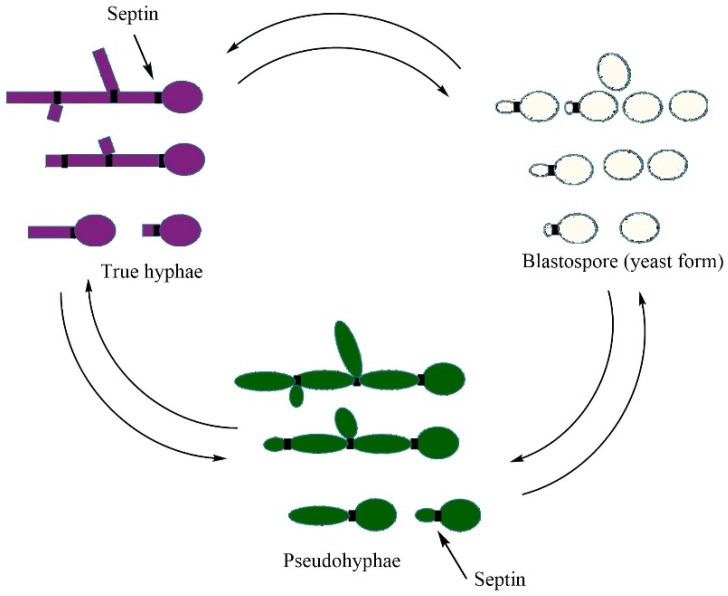
Morphogenetic transition cycle of *Candida albicans* showing the reversible transition between blastospore (yeast), pseudohypha, and true hypha forms. The diagram illustrates the sequential development and cellular elongation that defines the morphological plasticity of the ability of the fungus to colonize, invade, and persist in avian hosts.

*Candida albicans* is a pathogenic organism whose morphological switches and transitions play a key role in its infection cycle [102]. The adaptability of the morphogenesis of its blastospore (yeast), pseudohyphal, and hyphal forms is reversible and determinative of tissue invasion (Figure 2). The reversible morphological plasticity of *C. albicans* between blastospore (yeast), pseudohyphal, and hyphal forms (Figure 2) is considered a trait of adaptability that facilitates invasion and long-term colonization of host tissues [103].

During morphological changes in *C. albicans* driven by hyphal growth, septin rings serve as structural frameworks defining cell division sites in yeast and pseudohyphae, while in hyphae, they persist in stabilizing apical elongation at the germ tube base instead of actual cell division. Septin rings are involved in different processes during the morphological changes in *C. albicans* [104]. Figure 2 represents these morphological transitions, showing the sequential development from yeast to pseudohyphal and hyphal forms, with septin ring localizations (indicated by black transverse lines) that regulate cellular elongation, which is essential in tissue invasion.

**Figure 3:**
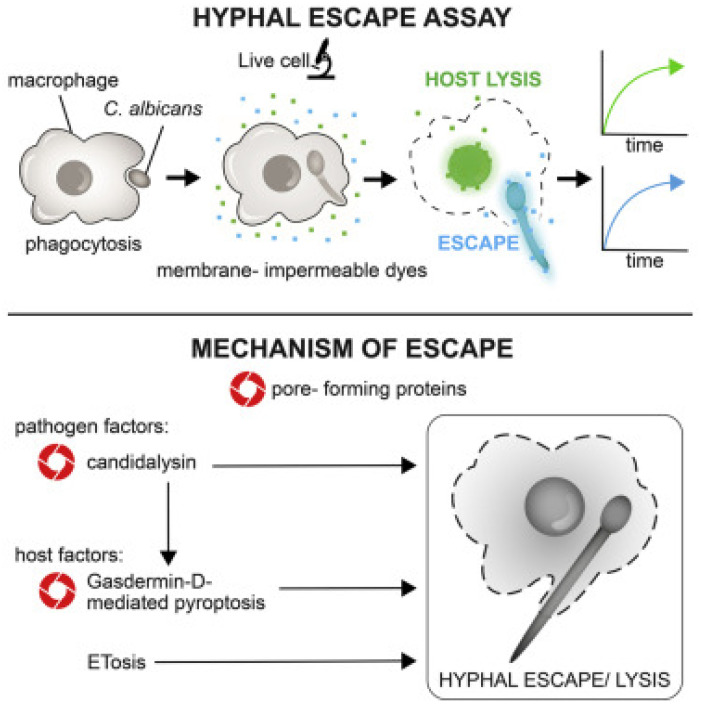
Mechanisms of hyphal escape and host cell lysis by *Candida albicans*. The hyphal escape assay is illustrated on the upper panel, with the *C. albicans* filaments growing long in phagocytic cells, rupturing membranes, and killing them. Lower panel describes the molecular and cellular processes that take place, such as membrane-permeable dye uptake, ETosis, and pyroptosis mediated by gasdermin-D caused by fungal and host factors. Reproduced from Olivier et al., Cell Reports, 2022, licensed under CC BY 4.0 [107].

Hyphae physically invade mucosa during infection and release virulent peptides, including Candidalysin, a product of the ECE1 (encodes endothelin-converting enzyme 1) gene that directly lyses epithelial cells and activates epithelial host danger-signaling pathways, including interleukin-1 family cytokines and inflammasome-mediated responses. Apart from epithelial invasion, hyphal growth makes *C. albicans* resistant to host defense cell interactions. When consumed by a neutrophil or a macrophage, the fungus may elongate across the phagosome, leading to membrane stress and rupture. It was demonstrated that Candidalysin, a cytolytic hypha-specific toxin, has a crucial role in candidiasis occurrence [103]. Candidalysin is a 31-amino acid alpha-helical amphipathic peptide synthesized by the hyphae of *C. albicans*. It has a significant function in the fungal defense machinery by damaging and destroying immune cell membranes of the host cell [105]. It is believed that it serves in instilling a systemic infection and mortality [106]. Candidalysin can directly injure epithelial tissue by permeabilization, perforation, and poration, leading to the weakening of the cytoplasmic contents [105]. A two-step process is involved, comprising Candidalysin assisting in the permeabilization of the membranes, in addition to activation of gasdermin D-mediated pyroptosis, stimulating the host lytic cell death. Moreover, ETosis (extracellular trap-mediated killing), another form of immune response, can take place, but *C. albicans* hyphae usually circumvent these traps via mechanical force and swift elongation. The concomitant occurrence of these events helps the fungus to escape phagocytic clearance and diffusion to the deeper tissues (Figure 3) [107].

The coordinated damage of epithelia and immune response contributes to local inflammation and additional invasion of host tissues by fungi. The regulators of this switch are EFG1, CPH1 (cyanobacterial phytochrome 1), RAS1 (Ras-like protein 1), and the cAMP-PKA and MAPK signaling axes. Hyphal forms cause mechanical pressure and release effectors that invade epithelium through induced endocytosis and active penetration to result in erosions, pseudomembranes, and caseous plaques typical of avian lesions [47]. These pathways have been reported in mammalian models and are highly conserved mechanisms involved in avian mucosal disease [47,108].

#### 2.3.3. Phenotypic Switching, Environmental Stimulus, and Enzyme Secretion

**Figure 4:**
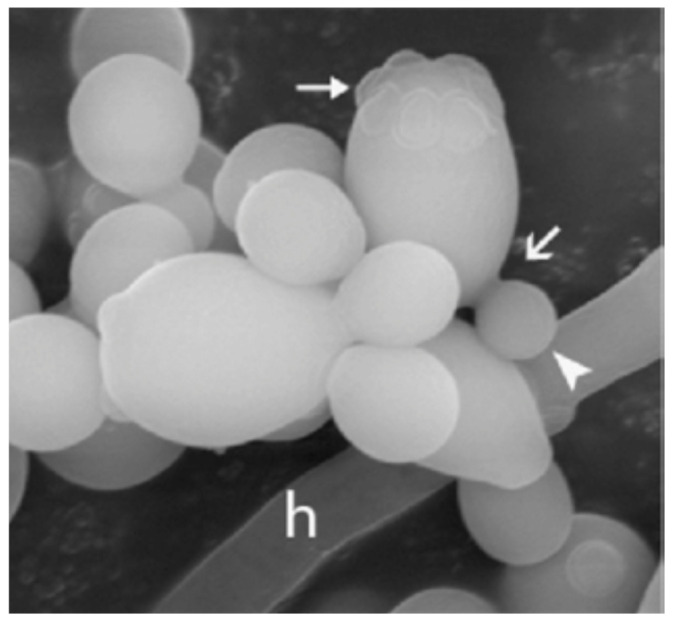
*Candida albicans* scanning electron micrograph (SEM) of yeast, budding, and hyphae. Arrows are used to denote budding cells and constriction sites of yeast, and h is used to denote emerging hyphae filaments, which is the morphological transition that is required in tissue invasion. Adapted from Staniszewska et al., Brazilian Journal of Microbiology, 2013, licensed under CC BY-NC 4.0 [109].

Originally, *Candida* reproduces asexually via the budding mechanism and, in response to environmental stimuli, transforms into hyphae, the elongated filamentous forms. Figure 4 [109] depicts the ultrastructural appearance of *Candida albicans* at the moment of morphological transition, i.e., the appearance of budding yeast cells still tied to the newly developed hyphal filaments, which reflect the invasive stage of the organism [12].

**Figure 5:**
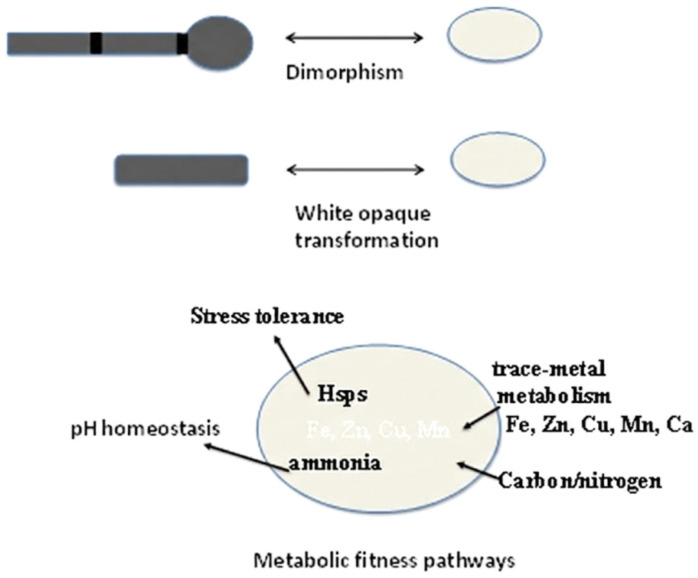
Dimorphism, white/opaque switching, and metabolic fitness adaptation (pH regulation, C/N, and metal homeostasis) are virulence characteristics of *Candida albicans*.

*Candida* albicans alters its benign yeast form into invasive pseudo-hyphae and true hyphae in response to environmental signals, including physiological temperatures (37 °C), pH fluctuations, nutrient status, high levels of CO_2_ (approximately 5 percent), and host immune signaling [110,111,112]. Once adhered, *Candida* exudes a variety of extracellular hydrolytic enzymes, including secreted aspartyl proteases (SAPs), agglutinin-like sequence ALS proteins, lipases, and phospholipases, disintegrating cell membranes and extracellular matrix components of the host cell [46,113,114]. These enzymes not only facilitate tissue invasion but also contribute to immune evasion by disrupting local immune signaling and impairing phagocytic activity [46,115]. Moreover, the genetic instability of *C. albicans* can modulate the *Candida* behavior with respect to growth rates, morphology, host interface, and stressor resistance, encompassing antifungals or antimicrobial peptides, as well as fungus pathogenicity throughout mucosal and systemic infective invasion [116,117]. As an example, *C. albicans* can switch its genetic pathways that sustain resistance to iron depletion in the bloodstream to those that sustain iron toxicity in the intestine during the commensal pathogenic transition [118]. This metabolic plasticity is closely connected with physiological and morphological suppleness. The capability of *C. albicans* to reversibly switch between yeast and filamentous (hyphae) forms of growth is known as dimorphism (Figure 5) and is necessary for invasion and tissue colonization. The organism also undergoes a white opaque transformation, a heritable phenotypic transition that changes mating potential, surface antigens, and immune interactions. Figure 5 combines some of these attributes, connecting dimorphism, switching, and metabolic fitness pathways. The latter involves heat shock proteins (Hsps) for stress tolerance, ammonia-mediated pH homeostasis, and carbon/nitrogen (C/N) and trace-metal (Fe, Zn, Cu, Mn) metabolism that favor fungal survival in various environments of the avian hosts.

#### 2.3.4. Secreted Hydrolytic Enzymes and Food Intake

Virulence is amplified by hydrolytic enzymes and proteins, especially SAPs, staphylococcal superantigen-like (SSL) proteins, and phospholipases, which degrade host proteins and membranes. Hydrolytic enzymes and proteins, particularly SAPs, staphylococcal superantigen-like (SSL) proteins, and phospholipases, amplify virulence by damaging host proteins and membranes, exposing ligands for adhesion as binding sites, and supplying peptides and lipids as nutrient sources in the nutrient-variable crop environment. SAPs are also capable of cleaving complement proteins and other innate immune effectors and help in immune evasion. SAPs also regulate host immunity, complementing interference, and promote the process of persistent infection [119,120]. By neutralizing the complement system, these microorganisms evade destruction by the immune system and become long-term colonizers [121,122]. Their survival in the crop microenvironment can also be achieved by iron-uptake systems (reductases, siderophore-related solutions) and metabolic plasticity (the ability to use different amounts of carbon available), thus overgrowing and persisting when bacterial competitors are suppressed [103].

#### 2.3.5. Biofilm Formation

**Figure 6:**
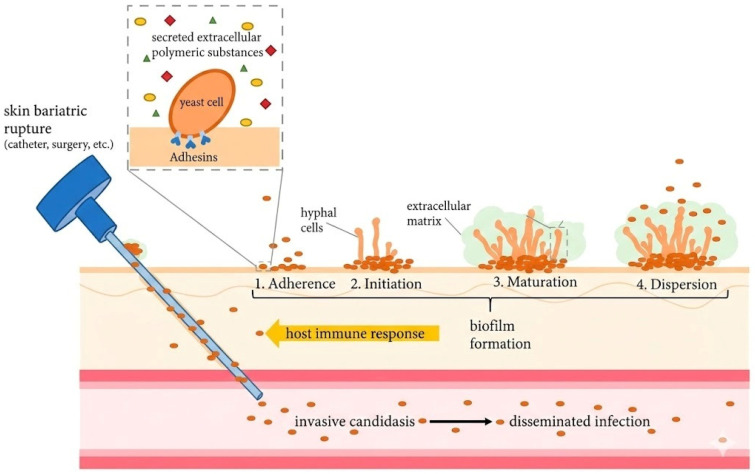
Sequential stages of *Candida* biofilm development on biotic and abiotic surfaces. Adapted from Amann et al., Pharmaceuticals, 2025, licensed under CC BY 4.0 [124].

BCR1 is a transcriptional regulator of various adhesin genes and is a key to effective biofilm development. Adhesion is the earliest stage in crop colonization, and formation of permanent surface communities or biofilms on mucosal surfaces and on feeding/drinking apparatus [114,123]. Figure 6 indicates the chronological stages of *Candida* biofilm development, which begins with cell adhesion to the biotic and abiotic surfaces and then develops to cell proliferation and the development of the hyphae. The thick interwoven network of hyphae is formed during the initiation and maturation stages into a protective fungal layer called the mycelia. It is followed by extracellular-matrix production, creating biofilm maturation, and the final dispersion step releases yeast cells, which may colonize new sites and contaminate medical equipment.

**Figure 7:**
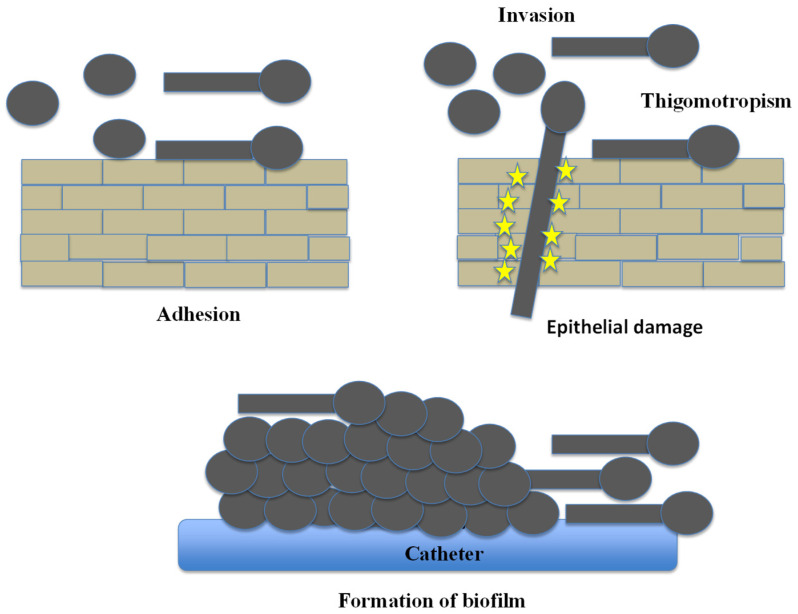
Sequential adhesion–biofilm invasion process of *Candida albicans*. The figure illustrates both epithelial adhesion or abiotic adhesion (e.g., catheters or feeding devices), biofilm formation, and hyphal invasion as a result of thigmotropism, engendering epithelial damage (yellow stars).

Though catheters are not frequently employed in avian patients, the schematic (Figure 6) depicts biofilm formation on medical devices similar to feeding tubes or syringes commonly used in psittacine and passerine hand-rearing and could be used as abiotic structures in these companion bird avian patients. Similarly, Figure 7 summarizes how the progress of initial adhesion of yeast cells yields multilayered biofilm formation. At the same time, directional extension growth response to physical contact, known as thigmotropism, guides the hyphal extension throughout epithelial surfaces for eventual invasion, inducing epithelial damage [125].

**Figure 8:**
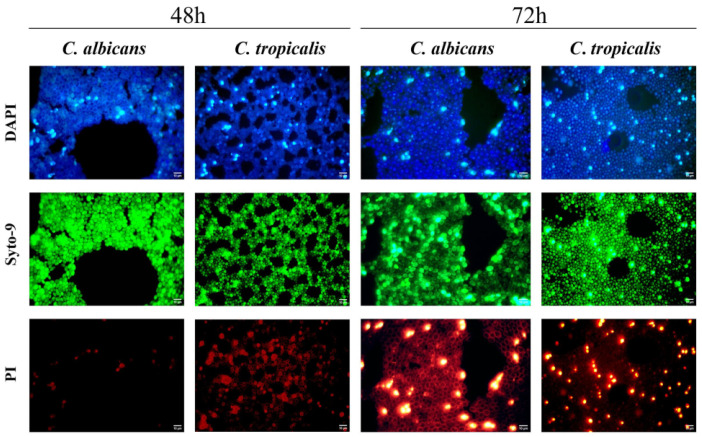
Illustration of the biofilms of *C. albicans* and *C. tropicalis* at 48 and 72 h of growth by epifluorescence microscopy using 4′,6-diamidino-2-phenylindole fluorescent stain and LIVE/DEAD Biofilm Viability Kit. Time samples of 48 and 72 h were used to compare the total cell and live/dead cells in the biofilms using an Olympus BX50 microscope, and pictures were obtained by AmScope software (v.10.11.2024) at ×100 magnification. Adapted from Atiencia-Carrera et al., Frontiers in Cellular and Infection Microbiology, 2022, licensed under CC BY 4.0 [126].

After initial adhesion to the mucosal surface, *Candida* cells establish mature biofilm communities, which present a significant clinical problem specifically in regard to drug resistance and treatment failure. *Candida* easily develops drug-resistant biofilms on avian mucosa and on indwelling or feeding apparatus (Figure 8).

Biofilms form a scaffold on which high-density growth occurs, limiting the ability of antifungals to penetrate the biofilm and protect hyphae and extracellular matrix elements, which are the basis of chronic or persistent crop infection [123,124]. Genes involved in adhesion, hyphal formation, and matrix production, such as BCR1, EFG1, HWP1, ALS3, and ECE1, are coordinately regulated during biofilm formation; mature biofilms produce an extracellular matrix that resists antifungal penetration and concentrates drug-resistant cells, which are responsible for determining treatment failures in chronic crop mycosis. Plasticity of biofilm architecture among strains and environments, such as abiotic and mucosal surfaces have been well described and is relevant to the translation of mammalian biofilm knowledge to the avian environment [123,127]. Crucially, the extracellular matrix acts as a diffusion barrier that confers resistance against common disinfectants [128], thereby allowing *Candida* to persist in poultry water lines despite standard sanitation protocols [129].

#### 2.3.6. Host Immune Response

**Figure 9:**
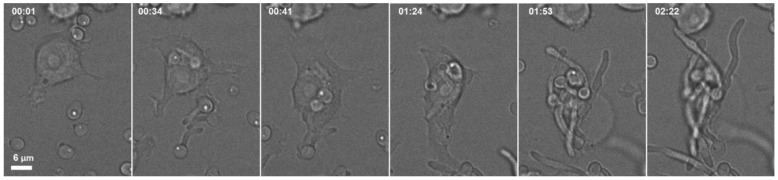
Sequential differential interference contrast (DIC) microscopy images illustrating the phagocytosis and subsequent lytic escape of *Candida albicans* from a macrophage. The progression is indicated by time stamps (hh:mm): (00:01) Initial recognition and engulfment of *C. albicans* yeast cells by the macrophage. (00:34) Complete internalization (phagocytosis) of at least five yeast cells; the fungal cells begin to undergo morphogenesis (yeast-to-hypha transition) within the phagosome. (00:41) Initiation of hyphal elongation is visible as the fungus attempts to expand within the host cell. (01:24) Continued hyphal extension exerts mechanical pressure, stretching the macrophage membrane. (01:53) The physical force of the extending hyphae leads to the rupture of the macrophage membrane. (02:22) Complete lysis of the macrophage and fungal escape, allowing for dissemination. *Candida* Reproduced from Bain et al., Seminars in Immunopathology, 2015, licensed under CC BY 4.0 [138].

The defense of the host against *Candida* infection is a dual mechanism that involves adaptive and innate immune cells [5,130,131]. Preserved epithelial barriers, antimicrobial peptides, and Th17-mediated responses are the defensive mechanisms of the mucosal surfaces. *Candida* avoids host immune defense by masking its cell wall, evading complement by binding to host regulatory proteins, proteolytic cleavage of complement components, and biofilm-mediated protection [120]. The *Candida* virulence pathways quantify and adapt to the host milieu at the same time; fungal PAMPS and candadysin activate the release of epithelial cell cytokines and generate innate effectors (neutrophils and heterophils), while proteolytic cleavage of host factors and fungal masking of 8-glucan inhibit recognition. Infections provoke a coordinated local immune response in birds characterized by mucosal thickening, epithelial erosion, and heavy infiltration of macrophages and heterophils, especially in the esophageal mucosa and the crop, which has demonstrated to decrease the pathogen content of the affected tissues [132,133,134]. These cells strive to contain infection through the promotion of phagocytosis and the release of reactive oxygen species, but the filamentous forms of *Candida* in avian hosts are more resistant to phagocytic killing [135]. This resistance is justified by the occurrence of rapid hypha elongation and branching of *Candida* spp., enabling the filaments to outgrow macrophage ingestion and making them too large to be phagocytosed [135]. Resultantly, when attempting to ingest hyphae, macrophages generate frustrated phagosomes, a phenomenon attributed to *Candida* spp., which utilize overabundant filamentous extensions to impede immune clearance [136,137]. The failed phagosomes are usually broken by the invasion of a hypha that physically tears the phagosomal membrane, leading to lysis of the macrophages and fungal growth [106,107,136,137]. *Candida albicans* hyphal/filamentous cells avoid and evade the phagocytes by lengthening and piercing through the host cell (Figure 9) [138].

**Figure 10:**
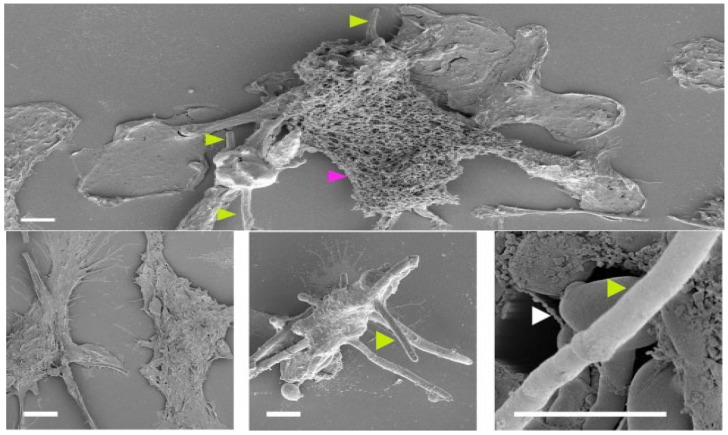
SEM images of Macrophage Extracellular Trap (MET) formation and hyphal escape. The (**upper panel**) displays a macrophage responding to fungal invasion; the pink arrowhead indicates the dense meshwork of hyperplastic extracellular traps (METs) on the cell surface, while green arrowheads point to *Candida albicans* hyphae penetrating and escaping the cell. The (**lower panels**) show uninfected macrophages (**left**), hyphal escape ((**center**), green arrowhead), and a high-magnification view (**right**) showing single MET fibers (white arrowhead) interacting with a hypha (green arrowhead). These MET structures trap but do not frequently kill the *C. albicans* hyphae, hence permitting the persistence of fungi in host tissues. Adapted from Olivier et al., Cell Reports, 2022, licensed under CC BY 4.0 [107].

Scanning electron micrographs (SEM) further reveal the formation of macrophage extracellular trap (MET) response to hypha invasion (Figure 10). In this process, macrophages react to immoderately long hyphae by releasing fibrous extracellular networks made up of DNA, histones, and antimicrobial proteins to create a mesh that entraps fungal filaments. Even though these MET structures have the potential to limit fungal invasion, *C. albicans* microorganisms tend to survive or evade them by further hyphal elongation and dichotomy, eventually undermining the integrity of phagocytes and promoting their survival inside host tissues (Figure 10) [107].

Although these panels are produced in mammalian systems, this mechanism (rapid hyphal elongation/branching to allow escape and resistance to killing) is widely conserved in the avian system [49]. Furthermore, histopathological studies exhibit massive invasion of inflammatory cells in the affected gastrointestinal coats, particularly macrophages with occasional eosinophils [139].

#### 2.3.7. Lesion Pattern and Progression

Infrequently, proximal lesions most often affect the esophagus and the crop with wrinkled and thickened mucosa covered by whitish raised plaques/pseudomembranes. Other analogous alterations may spread to the oral cavity and, less commonly, to the intestine and the proventriculus. Histologically, hyphae and pseudo-hyphae colonize the superficial mucosa with epithelial necrosis and mixed inflammation. Although rare, systemic colonization may occur in seriously afflicted birds [120]. Microscopic analysis of the depleted gosling is frequently characterized by growth of hyphae in the esophageal mucosa. The morphology of these gut lesions is consistently linked with a disruption of gut homeostasis caused by mold-contaminated food and antibiotic-generated flora deviations [89,140]. Such lesions tend to accelerate crop stasis and regurgitation, which can extend into a systemic disorder with weight loss and anorexia, features of the opportunistic pathogenic activity of *Candida* in the avian upper gastrointestinal tract [9].

#### 2.3.8. Etiologic Spectrum in Birds

Molecular epidemiological studies in poultry and waterfowl have supported the preference of the yeast for the upper gastrointestinal tract, specifically the crop and esophagus [120].

## 3. Diagnostic Strategies of Avian Candidiasis

Early diagnosis is primordial due to the wide range of clinical outcomes. Adequate diagnosis of avian candidiasis begins with symptoms and signs such as poor appetite, lethargy, weight loss, and regurgitation [60]. Nevertheless, a definitive diagnosis necessitates a combination of laboratory tests comprising clinical, cytological, cultural, histopathological, species identification, and molecular techniques since no single approach can trustfully differentiate between colonization and invasive malady [60]. More to the point, a combination of molecular and antifungal susceptibility testing helps to gain a better insight into the dynamics of the infection and contributes to the creation of specific treatment protocols [37].

### 3.1. Pathological and Clinical Signs of Avian Candidiasis

Clinical observations of candidiasis in avian species are associated with a spectrum of clinical manifestations, from mild respiratory distress to obvious gastrointestinal lesions. The symptoms are highly versatile and depend on a vast list of variables, including species of birds, age, condition of the immune system, nutrition, and volume of infection. They represent significant opportunistic fungal diseases in a variety of captive, domestic, and wild birds affected by suboptimal conditions and predisposing factors comprising corticosteroid treatment, stress, poor husbandry settings, malnutrition practices, or antibiotic overuse, all of which predispose birds to fungal overgrowth and subsequent tissue colonization. The signs are extremely variable and contingent on a wide range of factors such as species of birds, age, immune system, nutrition status, and infection volume [6,7,60,89].

#### 3.1.1. Early Stage of Clinical Manifestations (Nonspecific Signs)

**Figure 11:**
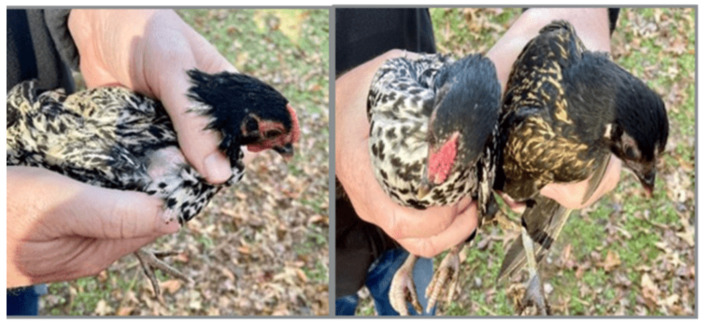
Backyard chicken (*Gallus gallus domesticus*) with pendulous crop (**left**) versus a normal bird (**right**). A non-specific sign is a crop distension that can occur due to impaction of feed, nerve damage, or overgrowth of secondary microbes (including *Candida* spp.). Note that differential diagnosis includes crop impaction, candidiasis, etc. Reproduced with permission from University of Maryland Extension (Photo: Jon Moyle) [143].

Early clinical manifestations of birds’ candidiasis are usually nonspecific and can even be confused with other disease conditions when observed physically. The case depicted in Figure 11 shows clinical manifestations for which the hypothesis of candidiasis has been made but still awaits confirmation. Definitive diagnosis requires laboratory confirmation by using either cytological examination, fungal culture, or histopathological essays, as described in the following Sections (Section 3.2, Section 3.3, Section 3.4 and Section 3.5). Symptoms in the initial stages are normally mild and of a non-specific nature because they can occur in many disorders, not just candidiasis, thus making it difficult to diagnose. The affected birds are likely to have decreased feeding habits, slight lethargy, occasional regurgitation, reduced vocalization, and slowed weight gain [60]. Elsewhere, early enteric dysfunction or cloacal irritation may be externally evident with soiling of the vent area by diarrhea or sluggish transit of the crop, which reveals the course of gastrointestinal imbalance related to *Candida* overgrowth.

The enteric type of manifestation is the most common, and the lesions are typically found in the crop and sometimes in the oral cavity, as the oral lesions can be present but not always visible, with the crop being the most consistently and seriously affected site. A significant distension of the crop is therefore observed, which is in line with crop stasis due to *Candida* infection of the crop mucosa (Figure 11). However, crop distension, in turn, is a non-specific clinical finding that can further occur from feed impaction and nerve injury, as well as secondary microbial imbalance, and hence should be interpreted with caution. In severe cases, the lesions can spread to the esophagus, proventriculus, and ventricle [141,142].

In addition to enteric manifestations, oral manifestations may appear in the initial stage of the disease. The emergence of whitish yellow, caseous pseudomembranes or plaque formations that adhere to the epithelium of the oral cavity, the tongue, or the palate is considered a typical, yet not pathogenic, sign of *Candida* overgrowth. Although these oral lesions represent a strong indication of thrush, cytological or histopathological tests would definitively determine the diagnosis of the disease, as similar lesions can be caused by other infectious or inflammatory occurrences.

#### 3.1.2. Gastrointestinal Upper Involvement

In cases where the oral cavity is infected, birds now tend to develop dysphagia, halitosis, regurgitation, and visible whitish plaques on the tongue and oral mucosa. These plaques may accumulate to create thick pseudomembranes of lesion compositions that may hamper food consumption and predispose tissues to secondary bacterial infections, thereby worsening inflammation and irritation [6]. The lesions may begin as minor lesions and eventually develop into devastating pseudomembrane and ulcerative lesions involving the palate and commissures [144].

#### 3.1.3. Gastrointestinal Tracts Gross Lesions (Crop, Esophagus, and Proventriculus)

The major infection site is the crop. Birds develop clinical stagnation in crop emptying, reduced crop fill, regurgitation, and noticeable crop distension. In normal birds, on the contrary, the mucosa of the crop is smooth and shiny with no apparent thickening or plaques. White-to-yellow adherent plaques or pseudo membranes, which can bleed when removed, are often discovered by oral examination of the mucosa. Gross lesions most often involve the crop, and the underlying mucosa is usually thickened and friable with whitish/grey pseudomembranes engendering the typical “Turkish towel” appearance [60,145].

In serious or chronic cases, the infection can spread to the esophagus and proventriculus, resulting in diarrhea, maldigestion, and vomiting [145].

#### 3.1.4. Susceptibility and Differences Related to Age

Young birds (both poultry chicks and hand-reared companion birds) are more susceptible to age-related factors as their immune system is still underdeveloped, and they are more vulnerable to environmental stressors. The disease, characterized by dehydration, the impaction of the crop, ruffled feathers, diarrhea, and retarded growth, progresses severely and rapidly in young poultry chicks and hand-reared psittacine and passerine nestlings and chicks [60]. By contrast, mature birds can show chronic weight loss, anorexia, lack of energy, reduced apparent mucosal lesions, and low egg production [4].

#### 3.1.5. Extra-Intestinal and Cutaneous Sign

Cutaneous candidiasis is not very prevalent but may correlate with the conditions of the trauma site or loss of feathers. Infected areas present rupture of feathers, which is associated with crusting dermatitis and hyperkeratosis. Comb candidiasis has been usually identified in poultry exhibiting erythematous lesions added to crusty scales around the wattles and the comb [6].

#### 3.1.6. Advanced Systemic Dissemination

Systemic implications might generate complex or widespread infections, usually witnessed in birds with concomitant diseases or those that are immunocompromised. Birds can be seriously debilitated with emaciation, intractable anorexia, persistent diarrhea, and malabsorption symptoms because of intestinal invasion. At late stages of the malady, the infection can spread via the esophagus, forming thickened mucosa and caseous or diphtheritic plaques, which can either ulcerate or coalesce [146,147]. In uncommon instances, the spread to the internal organs can cause coelomic effusion, splenomegaly, and hepatomegaly [145]. Proventricular involvement can also occur, presenting white-grey necrotic lesions and mucosa thickening that further troubles digestion and worsens the incapacitated state of the bird [147]. Effectively, affected birds might develop oral plaques, conjunctivitis, or stomatitis, which may additionally involve oral lesions mirroring superficial colonization of mucosal tissues in early localized disease stages. These pseudomembranes are histologically composed of necrotic epithelial cells, fibrin, and infiltrating leukocytes with hyphal invasion of the mucosa. Unfortunately, with the development of the infection, disseminated candidiasis can cause stagnation of crops, hepatosplenomegaly, respiratory distress, and coelomic fluid build-up, indicating the life-threatening invasive fungal infection of the internal organs [9,140,148]. In chronic diseases, chronic inflammation can cause crop wall thickening. Malabsorption of essential nutrients may occur, and beak abnormalities have also been reported in birds afflicted with candidiasis [5,6]. Additional frequent symptoms comprise esophageal lesions and severe pulmonary congestion, along with oral fungal thrush that can impair feeding and respiration [89,140]. Further abnormalities may include ocular complications such as fixed pupils or corneal swelling, anorexia, profuse diarrhea, profound weight loss, dehydration, and lethargy [148,149]. Clinically, systemic candidiasis is less prevalent; however, its mortality rate is high because it affects the vital organs [5]. Given the wide spectrum of clinical outcomes, the need to conduct diagnostic evaluation and medical treatment to reduce morbidity and mortality is crucial [150]. Therefore, early mycology culture and antifungal susceptibility testing remain important in order to direct the targeted therapy and enhance outcomes [19].

#### 3.1.7. Post Mortem Findings

**Figure 12:**
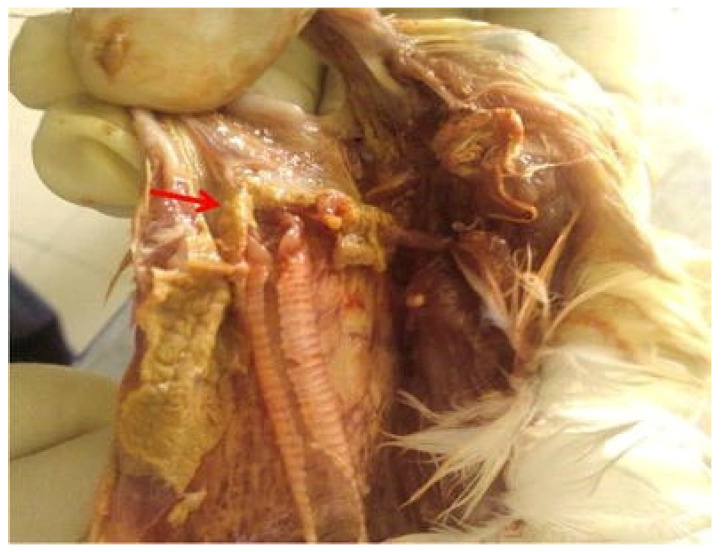
A yellowish-white pseudomembrane of the pharynx, larynx, and the proximal esophagus (red arrow). Pseudomembrane is a symptom of *Candida albicans*-infected necrotic mucosa, which is typical of avian candidiasis in pigeons (thrush). Reproduced from Mugale et al., Comparative Clinical Pathology, 2015, licensed under CC BY 4.0 [151].

In post mortem examinations of birds that died of candidiasis, pseudomembranes of a thick, yellowish-white character are usually evident in the crop, oropharynx, and proximal esophagus (Figure 12) [151,152].

**Figure 13:**
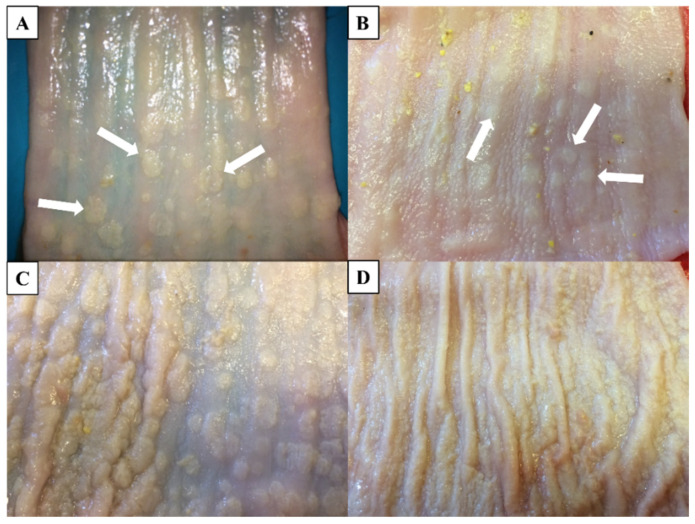
Yeasts producing different stages of mycosis that are characterized by whitish and thickened areas on the esophageal mucosa of geese. (**A**,**B**): Not severe esophageal mycosis; (**C**,**D**): severe esophageal mycosis. The yellowish-white lesions on the surface of the mucosa have different sizes, denoted by white arrows. Reproduced from Domán et al., Frontiers in Veterinary Science, 2023, Supplementary Figure S1 licensed under CC BY 4.0 [145].

In addition to lesions in the pharynx and proximal esophagus, a large amount of white-yellowish pseudomembrane can often be observed coating the esophageal mucosa, indicating extensive colonization and necrosis of the epithelial surface [152]. In necropsy, avian crop mycosis may display thick, caseous, yellow-white pseudomembranes that attach to the crop mucosa [153]. Characteristically, these diphtheritic membranes are typically readily peeled off, leaving behind an eroded, hyperemic mucosal lining below them. *Candida albicans* and its allied species have been reported to cause similar lesions in chickens, turkeys, pigeons, and psittacines [5,6,23]. In severe cases, the plaques can spread out of the mouth and the pharynx to the upper esophagus and sometimes become lumped together to block the lumen [145]. Figure 13 shows the successive phases of candidiasis affecting the esophagus mucosa as the illness progresses from moderate thickening with scattered white plaques and then moves to severe lesions with yellowish coalescent lesions and widespread mucosal necrosis [145].

### 3.2. Microscopic Analysis of the Cells

**Figure 14:**
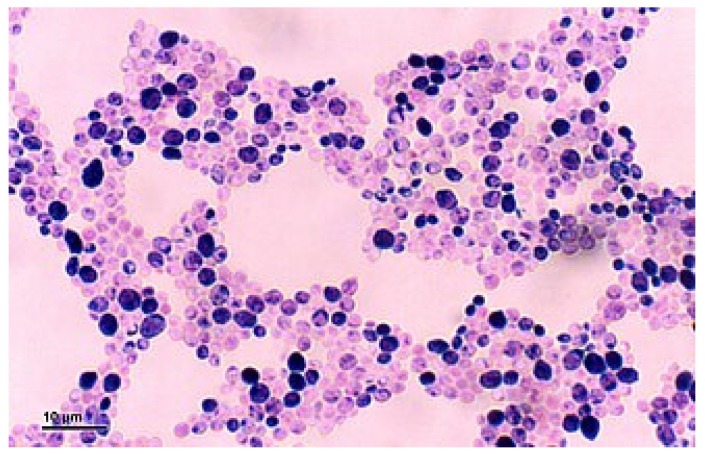
Light microscopic observation of *Candida* spp. with hundreds of rounded to oval-shaped budding yeast cells (blastoconidia) in loose clusters. The lack of pseudohyphae or true hyphae is associated with early colonization or a non-invasive/low-virulence form of candidiasis. Image by J. Reischig, licensed under CC BY-SA 3.0 [155].

Direct microscopic study of clinical samples (e.g., fecal samples, lesion scrapings, choanal swabs, crop swabs) or colony isolates is still at the core of diagnosis in birds [6,60]. Cytological examination of stained smears from the oral cavity, the conjunctiva, or the crop facilitates inexpensive and rapid visualization of yeast cells and hyphal microorganisms. This diagnostic technique allows direct screening but is not specific in differentiating incidental and pathogenic *Candida*. Therefore, molecular confirmation or complementary culture techniques should be performed to ascertain the existence of invasive *Candida* infection. Some of the commonly used stains include Diff-Quik, Giemsa (Romanowsky-type), Gram, or occasionally new methylene blue, which stains hyphae, pseudohyphae, or budding yeast cells [60,154]. Through light microscopic analysis of stained preparations, typical morphological characteristics of yeast cells emerge in the form of round to oval budding forms (blastoconidia) arranged as clusters (Figure 14).

**Figure 15:**
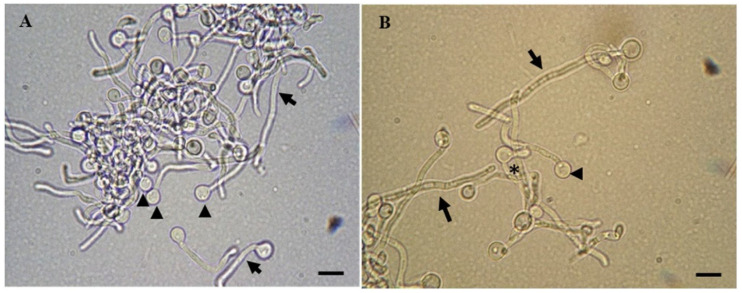
*Candida albicans* cells in cell transition analyzed by light microscopy. In (**A**), a combination of chlamydospores (arrowheads) and hyphae (arrows) is found. In (**B**), pseudohyphae (asterisk), chlamydospores (arrowhead), and multicellular hyphae (arrows) are seen. Scale bars represent 5 μm. Reproduced from Macias-Paz et al., Revista Argentina de Microbiología, 2023, licensed under CC BY-NC-ND 4.0 [114].

It is notable that chlamydospores, which are thick-walled resting spores, can be specifically identified in invasive cases and are observed under direct microscopy. Figure 15 shows morphological variation such as pseudohyphae, true hyphae, and chlamydospores in invasive *Candida* infection.

Particularly, the detection of hyphae validates tissue invasion rather than surface invasion. Wet mounts from scraped mucosal lining or by culture, on examination under light microscopy, reveal oval-shaped, budding yeast cells. Some fungi, like *Candida* species, appear Gram-positive when stained, therefore taking on the color purple or reddish-purple. This staining property is due to their cell wall structure since *Candida* has thick cell walls made not of peptidoglycan but of complex polysaccharides and glycoproteins, thus being able to retain the crystal violet primary stain post-decolorization, similarly to bacteria during the Gram staining stage [156,157]. In more invasive infections, *Candida* spp. might display pseudohyphae or true hyphae [158,159,160]. Although cytology is sensitive to superficial infections, it can furnish false negatives in deep mucosal disorders [6,60]. Thus, to confidently ascertain tissue invasion, complementary histopathologic special stains, like PAS or GMS, should be accomplished [161].

### 3.3. Culture Techniques

**Figure 16:**
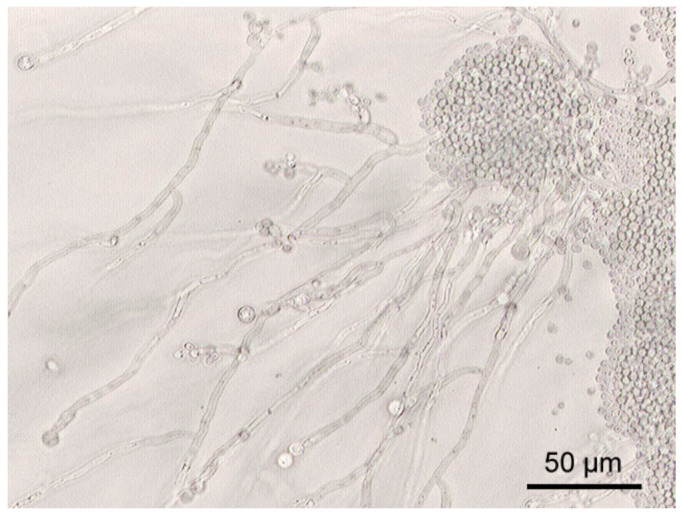
Microscopic photograph (200×) of *Candida* albicans ATCC 10231 on cornmeal agar plate containing 1 percent Tween 80, demonstrating blastospores (budding yeast cells), pseudohyphae, and chlamydospores. The occurrence of pseudohyphae and chlamydospores is an indicator of the transitional phase to invasive growth and virulence. Image by Y. Tambe, licensed under CC BY-SA 3.0 [166].

Although fungal culture continues to be the precise method for species susceptibility and identification test analysis, its prolonged incubation duration (usually up to 4 weeks) and low sensitivity suffers from utility limitations for timely avian candidiasis diagnosis [162,163]. Cultures are typically incubated at 37 °C for 48–120 h under aerobic conditions, after which characteristic colonies appear within a period of 48–120 h under aerobic conditions. In the majority of psittacine and passerine birds, *Candida albicans* colonies commonly appear as smooth, shiny, white-to-cream, dome-shaped colonies of 2–3 mm size after 2–5 days of incubation. Species differentiation based on colony morphology is limited; hence, further testing is necessary [164]. Besides colony morphology, microscopic evaluation of cultured *C. albicans* gives an understanding of the morphogenetic plasticity of the organism in response to changing physicochemical conditions. Sabouraud Dextrose Agar (SDA) is the most widely used primary culture medium in isolating *Candida* species because it allows growth of yeast due to its low acid pH and high dextrose concentration, and a large number of bacterial contaminants are suppressed [4,37,165]. Selective and chromogenic agar is commonly used in clinical practice, e.g., Sabouraud Gentamicin Chloramphenicol Agar (SGCA) [4] or CHROMID *Candida* Agar (CCA) [37], a trade name used to refer to a variant of CHROMagar *Candida* [165], to induce early differentiation of *Candida* spp. over other yeasts and to suppress bacterial and mold overgrowth. *Candida albicans* can also form typical blastospores, pseudohyphae, and chlamydospores on cornmeal agar with 1% Tween 80, which can be used to differentiate species at the species level and determine morphogenetic transition (Figure 16).

SGCA, mainly used to isolate and enumerate yeasts in highly bacteria-contaminated samples, is a selective medium that combines chloramphenicol with gentamicin to restrict the growth of other yeasts and bacteria and facilitates the premature segregation of *Candida* spp. [4]. CCA incorporates chromogenic substrates considered as yeast targets, inducing distinct colony colors for different *Candida* species, hence allowing presumptive species identification [37]. Culture also contributes to antifungal susceptibility testing as it is believed to be the most important criterion in therapy selection since antifungal resistance is on the rise in non-albicans *Candida* [3,6,167].

### 3.4. Species Identification

#### 3.4.1. Conventional Identification Procedures

Certain mycological culture media give diagnostic clues, specifically to *C. albicans*, which may be seen under microscopes. After 1 or 2 days of incubation at room temperature, *C. albicans* can develop chlamydospores or thick-walled asexual spores on Chlamydospore Agar or Corn Meal Agar that serve as diagnostic features [168,169]. In addition, the germ tube test is viewed as an inexpensive, fast method for the identification of *C. albicans*. In this method, colonies are inoculated with a little colony in avian or fetal calf serum and incubated at 37 °C for 2 to 3 h. *Candida albicans* is verified by the appearance of germ tubes as filamentous outgrowths lacking constriction at their base [170]. The tests are applicable in primary screening and presumptive identification, but they might not be adequate in identifying NACS and detecting mixed infections [171,172].

#### 3.4.2. Automated Species Confirmation and High-Tech Biochemical Methods

For a conclusive identification of a species, particularly in cases of antifungal resistance or unusual infections, biochemical and automated identification systems should be used. *Candida* species are highly identified using systems like API 20C AUX and VITEK 2 Compact, which utilize carbohydrate assimilation patterns or profiles of enzymes. Standardized, reproducible, and rapid results are achieved with these systems and allow accurate differentiation of *Candida* species. In avian medicine, such sophisticated procedures are of particular importance, as other NACS (e.g., *C. tropicalis*, *C. krusei*) can be intrinsically resistant to the popular antifungal agents like fluconazole, which requires proper identification to understand how to treat this problem [173,174].

### 3.5. Histopathology

**Figure 17:**
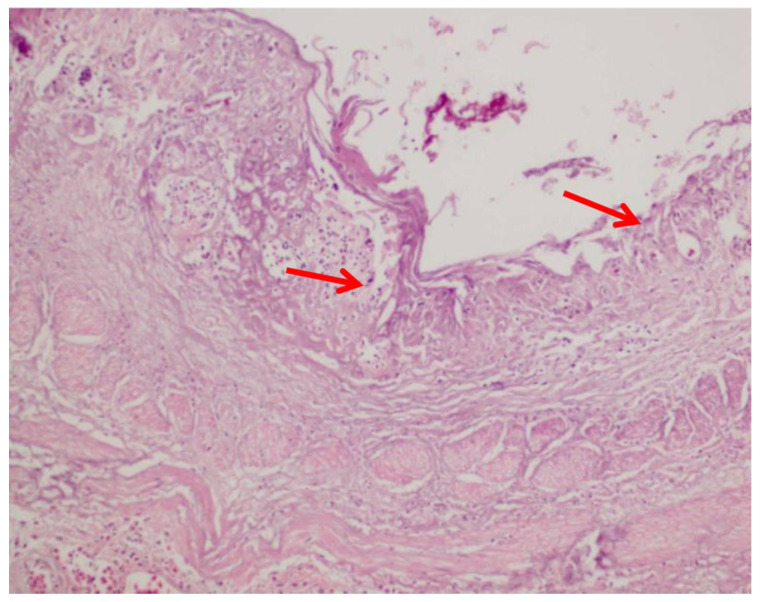
Histologic section of the esophagus revealing severe sloughing of the epithelium and inflammation (marked by arrows) in a pigeon with candidiasis. H&E staining. Reproduced from Mugale et al., Comparative Clinical Pathology, 2015, licensed under CC BY 4.0 [151].

Candidiasis has specific microscopic features that may be identified in post mortem histopathologic examination. After a conventional Hematoxylin and Eosin (H&E) staining procedure, epithelial hyperplasia, necrosis, and inflammatory exudate are demonstrated, whereas special stains are employed to visualize the fungal elements, such as periodic acid–Schiff (PAS) and Gomori methenamine silver (GMS). Histological analysis of damaged tissues comprising the crop, esophagus, and proventriculus provides the most definitive evidence of invasive candidiasis as a crucial indicator of systemic candidiasis, particularly in immunocompromised birds. Figure 17 illustrates significant necrosis and sloughing of esophageal mucosal epithelium with pyogranulomatous inflammation, which are usually depicted in histopathological features of candidiasis infection.

**Figure 18:**
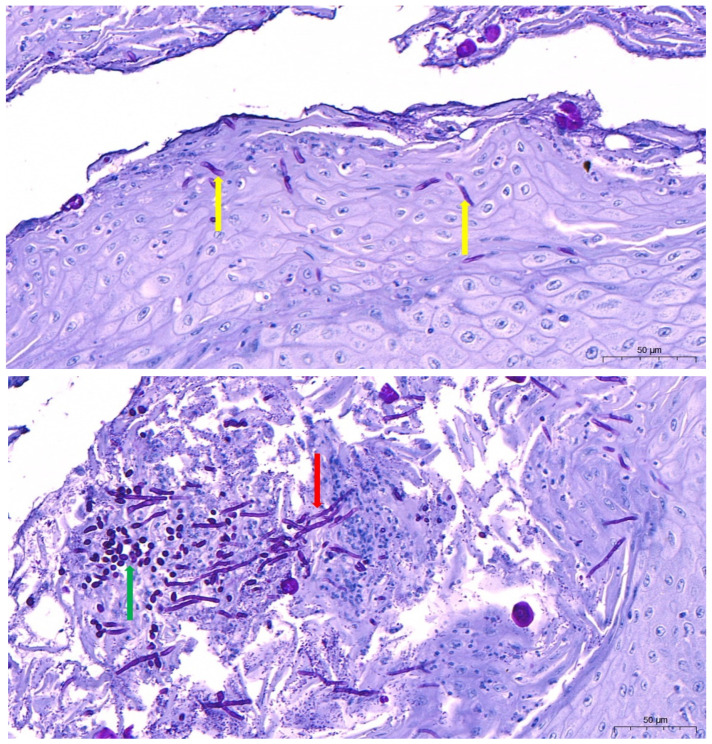
Histopathologic slide prepared from goose esophagus. The yellow arrows in the upper picture shows that pseudohyphae are present in the esophagus hyperplastic epithelium, the red arrow in the lower picture shows the presence of fungal hyphae in the esophagus, and the green arrow shows the presence of yeast cells in the esophagus. Periodic acid–Schiff (PAS) staining, 90×, bar = 50 µm 102×, bar = 50 µm. Adapted from Domán et al., Frontiers in Veterinary Science, 2023, licensed under CC BY 4.0 [145].

Histopathological examination tends to disclose the presence of budding yeast cells that are trapped during granulomatous inflammation or suppurative inflammation with hyphae invasion and tissue necrosis, evidencing the presence of systemic candidiasis [140]. In order to identify candidiasis, special tests are conducted, e.g., GMS and PAS stains. PAS stain identifies fungal factors in bright pink to magenta, whereas GMS shows them in black. The histopathological essays reveal mucosal necrosis, erosion of epithelial cells, infiltration of macrophages and heterophils, and hyphae in deeper tissues. Representative histopathologic observations are depicted in Figure 18, illustrating the presence of pseudohyphae in the hyperplastic esophageal epithelium with fungal hyphae and yeast cells infiltrating necrotic tissue as observed using PAS staining [145]. This is an important step that distinguishes between inoffensive colonization and true infection [3].

### 3.6. Serological Diagnostic Methods

Serological tests identify fungal infections by either revealing fungal antigens, as components of the microorganism, or host antibodies engendered by the immune system to fight the pathogens in response to infections [175]. Serological tests like neutralization and hemagglutination-inhibition (HI) tests are not the most common tests that identify antibodies against *Candida* in the avian species, since the tests are specific to viruses and not to fungi such as *Candida*. While HI is applied to identify the presence of antibodies against viruses by determining the level of antibody to prevent clumping of red blood cells, *Candida* tests typically require identifying antigens or the presence of antibodies against certain elements of the fungus [176]. Alternatives to HI or neutralization tests are the Mannan Antigen Test (MAT) and Enzyme-Linked Immunosorbent Assay (ELISA). Such methods can identify *Candida* antibodies in human beings and possibly in avian groups as well. MAT identifies mannan, a carbohydrate on the cell wall of *Candida* species, as it is a major diagnostic clue of invasive candidiasis [177]. ELISA is one of the most useful and sensitive tests that can detect the presence of specific anti-*Candida* antibodies or absent fungal antigens, helping to diagnose candidiasis, particularly invasive candidiasis. ELISA technology is a diagnostic method of identifying and quantifying a certain antigen or antibody in a sample by using a secondary antibody that is linked with an enzyme to generate a measurable coloration that is proportional to the quantity of the target present [178]. Even though the ELISA test can more or less be used to identify the presence of antibodies, the positive test should not be used to rule out a false negative, whereby the bird is fatally sick and is unable to produce an adequate immune response. Clinical signs, postmortem examination, and culture of the fungus of the affected tissues are the normal basis of the diagnosis [179,180]. In veterinary laboratories, immunodiffusion assays such as double immunodiffusion or counter-immunoelectrophoresis are usually accomplished owing to their reproducibility and specificity. These assays are able to detect anti-mannan antibodies or mannan antigens, which are considered to be one of the most important indicators of active candidiasis [181,182]. However, in birds, serological reactions are shaped by disease chronicity, species variations, and their immunity status [183,184]. Thus, serological tests are to be used together with clinical examination and culture or molecular outcomes [185].

### 3.7. Molecular Diagnostic Techniques

The use of molecular diagnostic techniques is now an inseparable part of prompt, sensitive, and specific detection of *Candida* species in avian medicine when low fungal burdens, non-culturable strains, or mixed infections complicate the use of conventional diagnostic methods [186,187,188]. Polymerase chain reaction (PCR) is the best-utilized molecular method in avian mycology [189]. It allows amplification of *Candida* DNA in diverse types of clinical samples, such as crop swabs, tissue biopsies, blood, and fecal material [190]. These techniques are used in supplement to traditional culture and microscopy, which gives unambiguous identification and helps in the promotion of antifungal therapy in time. PCR-based methods of *Candida* DNA detection in blood samples have demonstrated a higher sensitivity than other diagnostic methods [191]. PCR tests are traditionally developed to identify two categories of genetic markers, which would each have a different diagnostic role. The first category includes conserved ribosomal DNA sequences, i.e., 28S rRNA, ITS 1 -5.8S-ITS2, and 18S rRNA, which involve highly conserved sequences that allow the screening and identification of wide and diverse microbial communities at a genus level to identify *Candida* spp. [192]. The second class implicates species-specific genes such as HWP1(Hyphae Wall Protein 1), SAP (Secreted Aspartyl Proteinase), or ACT1 (Actin). These make it possible to differentiate species, separating *C. krusei*, *C. glabrata*, *C. albicans*, *C. tropicalis*, and other clinically important species [193,194,195]. Since *Candida* spp. tend to co-exist with other yeasts or bacteria in the gastrointestinal tract of birds, the high specificity of PCR is invaluable in establishing active infection over commensalism [40].

### 3.8. Advanced Diagnostic Techniques

Matrix-assisted laser desorption–ionization time-of-flight mass spectrometry (MALDI-TOF MS) furnishes a fast, species-level *Candida* isolate identification and, through combination with PCR-based assays of azole-resistance genes, provides a potent method of advanced fungal diagnostics [196,197]. The introduction of MALDI TOF MS to carry out rapid identification at the species level, assisted by PCR assays to identify azole resistance marks, could have a major impact on reducing diagnostic times [198,199,200]. However, since antigen-based assays have a short half-life in the blood, they must be thoroughly field tested to determine that lateral flow assays have adequate sensitivity in the avian host [201]. The promptness of MALDI TOF MS in generating species identifications within minutes, along with the capability of real-time PCR detecting resistance determinants, therefore provides a viable point of care solution to avian clinics requiring immediate and genotype-guided care [202,203]. Recent veterinary studies revealed that MALDI-TOF MS is efficient in recognizing within minutes avian *Candida* isolates, enhancing its appropriateness to fast diagnostic processes [201,204]. Other advanced molecular approaches implicate high-resolution molecular techniques such as real-time quantitative PCR (qPCR) or multiplex PCR that can be utilized to quantify fungal DNA or to concomitantly detect multiple *Candida* species in the case of mixed fungal infections in avian environments [205,206,207]. These tests provide high sensitivity of analysis and can be performed with short turnaround times that make them particularly beneficial in the detection of systemic or early-stage infections. PCR amplifications that produce DNA sequences, specifically the internal transcribed spacer (ITS) region, are considered the gold standard of the precise identification of species. Sequencing validates PCR data, identifies new or emerging NACS), and helps in molecular epidemiology and in monitoring antifungal resistance [208]. Next-generation sequencing (NGS) and metagenomic methods are being extensively investigated to describe the whole fungal microbiome of the avian gastrointestinal tract and to detect co-infections that may modify the development of a disease [209,210].

## 4. Prevention, Treatment, and Control of Avian Candidiasis

### 4.1. Prevention and Control

Routine prevention of avian candidiasis is based on the two main principles: (1) provision of adequate sanitary conditions at all times and (2) reduction in predisposing factors (immunosuppression, excessive use of antibiotics, and poor nutrition) that promote fungal growth [92]. Housecleaning, drinking, feeding, and hand-rearing equipment such as water lines (in commercial poultry), feeding tubes, and syringes (in psittacine and passerine hand-rearing) should be properly hygienic, as *Candida* can readily develop resilient biofilms within poultry drinking-water lines and on abiotic surfaces, thus presenting an ongoing infection foci and a persistent contaminated environment [6,123,129,211]. Major preventive strategies adherent to the management of fungal infections in flocks include daily cleaning of drinkers and feeders, appropriate feed storage, and regular disinfection using tested fungicides against yeast biofilms [129,212]. Moreover, biofilm-specific sanitation practices should be initiated in feeding systems, including mechanical cleaning and enzyme detergents, as an attempt of minimizing infection risk [213]. Importantly, impeding disturbance of the normal commensal bacterial flora is imperative by avoiding long courses or unjustified treatment using antibiotics that may cause overgrowth of *Candida* species [6]. Nutritional management, including the presence of sufficient levels of proteins and vitamins, particularly A and E, assists in mucosal immunity, while maintaining an optimal environment, including ventilation, humidity, temperature, etc., helps to prevent immunosuppression under stress [6]. Contaminated birds must be confined at once to prevent horizontal infection, especially in the case of a crowded commercial poultry flock or hand-reared psittacine and passerine population. Moreover, nesting areas should keep a check on the potential vertical pathway of transmission since fungal spores can be passed on in infected nesting material and eggshells [6]. To prevent contamination of eggshell, iodine or quaternary ammonium compounds can be dipped or sprayed on fertile eggs in order to lessen transovarial or eggshell-mediated infection of hatchlings. Flocks prone to recurrent crop infections should be regularly and closely monitored and subjected to frequent cytological, culture, or molecular screening, especially in flocks with recurrent crop candidiasis, since early diagnosis enables timely treatment before development of clinical symptoms such as regurgitation, plaque formation, and crop stasis [214]. The reports of waterfowl and poultry cases bring into the limelight the need to investigate feed cases and esophageal lesions in the occurrence of die-offs [6,212]. To aid in the prevention of clinical disease outbreaks, regular culture or molecular screening of *Candida* species should be incorporated into the regular management of flocks and breeding operations to detect sub-clinical colonization at its initial stages [215]. Last but not least, reducing the flock density, preventing abrupt environmental alterations, and ensuring convenient ventilation contribute to the reduction in stress [216,217].

### 4.2. Treatment

Basic parts of the treatment of candidiasis in birds are grounded in the inhibition of the underlying factors and implementation of the supportive treatment together with the antifungal therapy. A useful management strategy is aimed at therapeutic intervention and preventing factors of recurrence (dietary imbalance, long-course antibiotics, and poor hygiene).

#### 4.2.1. Interventions on the Environment and Management

Prevention strategies are aimed at improving husbandry and biosecurity by sterilization, routine disinfection of feeding tubes, optimum hygiene, or replacement of contaminated syringes in psittacine and passerine hand-rearing operations [218]. Initiating biofilm-specific sanitation practices in the feeding systems, such as mechanical cleaning and enzymatic detergents, is essential to reduce the risk of infection [213]. Additionally, reducing flock density, preventing abrupt environmental alterations, and ensuring convenient ventilation contribute to a reduction in stress [216,217].

#### 4.2.2. Antifungal Therapy

Nystatin (indicated dosages in pet birds: 300,000 IU/kg orally, twice a day) is regarded as the most appropriate drug to treat avian candidiasis because of low gastrointestinal absorption, low toxicity, and efficient potency against *Candida albicans* [60]. The use of azole antifungals, such as voriconazole, ketoconazole, fluconazole, or itraconazole, can be recommended as alternatives in the situation of proven resistance to nystatin or poor response. Hepatic toxicity, however, must be monitored with care, especially in the species of psittacine, which are highly sensitive to the azole compounds [219]. Among the azole family, azole derivatives are the most widely used antifungal agents available in the market with ideal pharmacokinetic characteristics and a broad spectrum of activity. Their antifungal effect implies disrupting the function and integrity of the fungal cell membrane. Effectively, they block lanosterol 14α-demethylase, a cytochrome-dependent enzyme in one of the significant stages of ergosterol synthesis, leading to ergosterol exhaustion and accumulation of toxic sterol intermediates, which muddle membrane structure and function [220]. Ketoconazole was the first orally effective azole-based antibiotic that has proved its effectiveness in systemic and local *Candida* spp. infections. Its supplementation (general dosage: 10–30 mg/kg orally, 2 times a day) in drinking water or food reduces clinical lesions and fungal load. It is, however, limited in its clinical use due to poor gastrointestinal absorption and risk of hepatotoxicity, which restricts its frequent use in avian species [220,221]. A second-generation triazole, Itraconazole (overall dose: 5–10 mg/kg, by mouth, once to twice daily, 7–14 days), was found to be more potent against azole-resistant *Candida* strains and better absorbed orally. Itraconazole is equipped to treat mucosal and systemic fungi in birds because of its capacity to penetrate tissues. Very recent studies demonstrate its superiority and decreased toxicity when used to manage avian mycoses compared to ketoconazole [222,223,224]. Fluconazole (5 to 20 mg/kg, oral route, every 24 to 48 h) (ref review Garcia) is another triazole antifungal that is well characterized by good oral absorption and bioavailability, water solubility, and a low toxicity profile [6,219]. It has shown potent inhibitory action in the case of *C. albicans* isolates in poultry and has been successfully utilized in the treatment of intestinal and oral *C. albicans*. Fluconazole (indicated dosages: 20 mg/kg, oral route, every 48 h) is considered the treatment of choice in cases where the yeast is resistant to nystatin in large avian flocks owing to its pharmacokinetic safety, stability, and convenience of administration [60,225,226]. Gentian violet (topical route) can be administered to the lesions of the mouth, like those caused by oral thrush, though precaution is demanded due to undesirable reactions such as mucosa irritation [60].

#### 4.2.3. Fungal Susceptibility Testing

**Figure 19:**
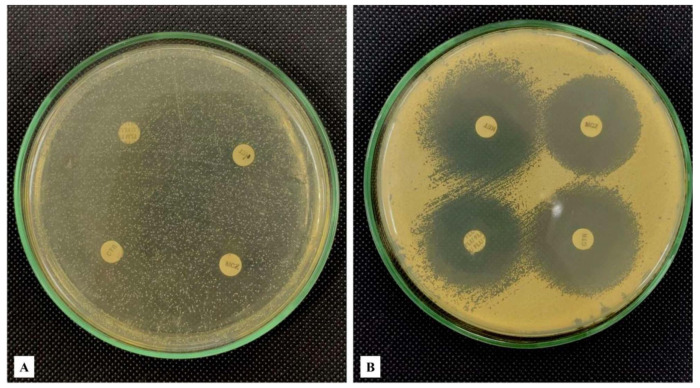
Antifungal susceptibility profile of *Candida* spp. isolated from the oral and cloacal cavities of parrots. (**A**) *Candida glabrata* isolates resistant to all 4 antifungals. (**B**) Isolate of *Candida albicans* with ketoconazole (upper left), miconazole (upper right), fluconazole (lower left), and clotrimazole (lower right) inhibition zone demonstrated. Reproduced with permission from Lima da Rocha et al., Journal of Avian Medicine and Surgery, 2025 [37].

In the case of emergence of azole-resistant infections in *C. albicans* and NACS such as *C. krusei* or *C. glabrata*, antifungal susceptibility testing (AST) is recommended before extended treatment. Reported studies have shown that both amphotericin B and nystatin are highly potent against AST [4,40]. The Clinical Laboratory and Standards Institute (CLSI) provides standardized guidelines for broth microdilution testing to evaluate yeast susceptibility to antifungal agents [227]. Several studies have investigated antifungal susceptibility patterns in avian *Candida* isolates. Berg et al. [228] described a case of *C. glabrata proventriculitis* in an eclectus parrot, which was successfully cured with nystatin and fluconazole. Antifungal susceptibility testing revealed that the lowest concentrations of nystatin and fluconazole that prevented the growth of the isolate were 2 and 32 μg/mL, respectively. Although the increased fluconazole concentration is a sign of possible resistance, the bird had a good reaction to the combination therapy, which highlights the necessity of exercising caution when applying the in vitro susceptibility results to clinical practice. Antifungal susceptibility of 84 *C. albicans* and 17 *C. catenulata* strains isolated in laying hens was studied by Rhimi et al. using broth microdilution testing [229]. The tested drugs include amphotericin B (AmB), fluconazole (FLC), itraconazole (ITZ), voriconazole (VOR), posaconazole (POS), micafungin (MCF), and anidulafungin (ANI). All *C. albicans* strains had high MIC values for azoles and AmB, and 22 strains were multidrug resistant to FLC, ITZ, VOR, and POS. It is interesting to note that all *C. catenulata* strains from eggs were ITZ-, POS-, MCF-, and ANI-resistant but AmB-sensitive. The resistance patterns differed depending on the source of isolation (cloacae, faeces, or eggs), indicating that environmental factors could determine antifungal resistance patterns. Mgbeahuruike et al. [230] examined *Candida* species in poultry environments in Nigeria and evaluated their susceptibility to nystatin (100 μg/mL), fluconazole (25 μg/mL), and voriconazole (1 μg/mL). *Candida parapsilosis* was sensitive to the three antifungal agents, and the areas of inhibition were 19.08–25.36 mm. Nevertheless, all Aspergillus species were found to be resistant to fluconazole but sensitive to nystatin and voriconazole. Other fungal isolates such as Penicillium, Mucor, Rhizopus, and Rhodotorula were also resistant to fluconazole and voriconazole but sensitive to nystatin. These findings underscore the fact that nystatin presented the widest range of activity against the evaluated poultry farm fungi. Lima da Rocha et al. [37] compared the antifungal susceptibility profiles of 12 *Candida* spp. isolates in the cloacal and oral cavity of 20 pet parrots to four azole antifungals (ketoconazole, fluconazole, miconazole, and clotrimazole) (Figure 19). Most *Candida* spp. isolates (58% 7/12) exhibited intermediate sensitivity or resistance to these drugs when used with fluconazole, having the lowest activity as an antifungal agent against *Candida* spp. in vitro. Among the intermediate susceptible species, 20 (1/5) were intermediate susceptible to clotrimazole, 20 (1/5) to miconazole, and 60 (3/5) to fluconazole. Of the species found to be resistant, 25% (2/8) were resistant to ketoconazole, 12.5% (1/8) were resistant to clotrimazole, 12.5% (1/8) were resistant to miconazole, and 50% (4/8) were resistant to fluconazole. Vieira and Coutinho characterized *Candida* species isolated from the crop of parrots (*Amazona* spp.) and identified five species: *C. humicola* (28%), *C. parapsilosis* (24%), *C. guilliermondii* (20%), *C. famata* (20%), and *C. albicans* (8%). Despite the fact that the study did not specifically assess antifungal susceptibility, the authors stated that species-level identification is of utmost importance in terms of attaining successful treatment effects, owing to the variability in susceptibility profile that is evident among various species of *Candida* [231]. The molecular identification and antifungal susceptibility test of *Candida* isolates of domestic chicken in Iraq were conducted using the Vitek 2 automated system by Kadhim et al. [38]. The study demonstrated that all isolates were susceptible to major classes of the tested antifungals, such as amphotericin B, fluconazole, flucytosine, voriconazole, caspofungin, and micafungin, indicating a lack of antifungal resistance of the avian *Candida* isolates in the present study [38]. The reported studies have shown that both amphotericin B and nystatin are highly potent against avian *Candida* isolates in many cases. Nonetheless, the inconsistency in the susceptibility trends among different studies, geographic locations, and origins of isolation (companion birds vs. poultry) expresses the necessity of regular AST in treating persistent or recurrent candidiasis in birds. Most species of NACS were not as susceptible to the studied azole antifungals. Table 2 summarizes the antifungal susceptibility data from avian *Candida* isolates.

These results are an important indication of the microbiota of birds and the potential development of the NACS resistant to azole antifungals that are widely used in human and veterinary practice. The resistance to *Candida* spp. identified in birds represents a problem in the One Health system, as the birds may act as reservoirs with drug-resistant yeast that can be passed onto a human via direct contact or environmental pollution. Poultry and companion bird breeding and handling can have significant human and animal health implications. To understand the chain of infection of the candidiasis, the mechanisms of the development of antifungal resistance in avian isolates, and the potential danger the resistant isolates have on human health, further research is needed [37]. A recent pilot study using comparative genome analysis demonstrated loss of heterozygosity in genes associated with azole resistance (*ERG11*, ERGosterol 11; *MDR1*, MultiDrug Resistance 1; *TAC1*, Transcriptional Activator *Candida* 1) in both human and avian *C. albicans* isolates, suggesting that environmental selective pressures may contribute to the spread of potentially resistant strains between hosts [232].

#### 4.2.4. Supportive Care/Management

Supportive care is necessary to enhance recovery and decrease mortality in birds infected with candidiasis because such birds are likely to present with dehydration, malnutrition, and crop stasis. Supportive care entails interventions aimed at treating crop stasis, which can be resolved by gentle emptying of the crop followed by offering smaller, more frequent feedings until normal gastrointestinal motility is reestablished [233,234,235]. Prokinetic agents, including metoclopramide (administered at 0.5–1.5 mg/kg orally or intramuscularly every 8–12 h) or cisapride (administered at the same dose and route at 8 h intervals), are recommended to increase crop motility and overcome regurgitation in persistent crop stasis [235,236]. Fluid therapy and proper nutrition are inseparable ingredients for the treatment of avian patients suffering from anorexia or dehydration. Fluid replacement is applied to replenish hydration and replace electrolytes, and it is preferably carried out using warmed isotonic fluids given orally or subcutaneously based on the severity [237]. Rectifying dietary imbalances via efficient nutritional support, particularly through high-protein food that is easy to digest (e.g., hand-feeding formulas for psittacines and passerines, or appropriate starter feeds for poultry) supplemented with sufficient levels of vitamins, such as A and E, is significant for the repair of mucosal integrity and immune competence. Addressing cases of concurrent infections like parasitic or bacterial disease and treating deficiencies in the body by proper supplementation can go a long way in helping in the healing process and even boosting the immune function [238]. Management is aimed at the treatment of underlying physiological imbalances and improvement of gastrointestinal activity. It is noteworthy that stasis does not recur in hand-reared psittacine and passerine neonates, as careful feeding practices and smaller meals at increased frequency can help in preventing recurrence. Supportive care thus plays a major role in conjunction with antifungal therapy to ensure complete resolution and avoidance of infection relapse [3,6].

### 4.3. Prognosis and Follow-Up

Localized crop candidiasis has a favorable prognosis with the implementation of early diagnosis and proper treatment. Persistent or systemic infections, especially in immunocompromised or newborn birds such as poultry chicks or hand-reared psittacine neonates, may require long-term treatment and have prognostic results [239,240]. The success of therapy (crop swabs and cytology) is ensured by re-examination [241]. Recurrence is usually an indication of continuous environmental pollution or unaddressed predisposing causes [4].

### 4.4. Existing Knowledge Gaps in Avian Candidiasis Research and Control

Despite increased avian candidiasis knowledge in the recent past, substantial gaps in knowledge persist that limit the effective control and preventive interventions [242]. To begin with, it is evident that there is a lack of avian-specific antifungal dosing regimens. The majority of treatment regimens are based on mammalian studies or on clinical experience gained empirically as opposed to pharmacokinetic data obtained in controlled clinical trials on birds [243]. Such a deficiency in species-specific dosing data can potentially lead to inadequate therapeutic efficacy or potential toxicity, especially with regard to the differences in physiological specificities in the metabolism of drugs in avian and mammalian species [219]. Second, the molecular nature of species-specific virulence factors of *Candida* isolates in the avian system is still not well understood [244]. At present, much has been carried out to explain the virulence mechanisms in human clinical isolates. While the epidemiology of bird yeast species has been thoroughly studied, the virulence profile of isolated species is not well investigated [244]. Furthermore, it is not known whether avian *Candida* strains express varying virulence factor profiles that can guide specific therapeutic measures [145]. Third, the genetic relationship between avian and human *Candida* isolates remains poorly characterized. One study using multilocus sequence typing (MLST) to compare *C. dubliniensis* isolates from seabird excreta in Ireland with human clinical isolates found that 13 of 14 avian-associated isolates were genetically distinct, forming a separate subgroup within clade C1, with six novel diploid sequence types (DSTs) and two new exZWF1b (glucose-6-phosphate dehydrogenase gene) alleles identified exclusively in avian strains; only one avian isolate was indistinguishable from human isolates, suggesting that cross-species transmission, when it occurs, may be primarily from humans to birds rather than vice versa [245]. Further molecular epidemiological studies are needed to clarify the direction and frequency of interspecies transmission of pathogenic *Candida* species. Fourth, since *Candida* spp. are opportunistic pathogens commonly present as commensals in the gastrointestinal tract of healthy birds, improved diagnostic approaches are needed to reliably differentiate between commensal carriage and clinically significant infection [5,49]. The existing diagnosis methods usually require laboratory facilities and have prolonged turnaround times, which can delay proper therapeutic intervention in commercial poultry operations or wildlife settings [5]. Fifth, the lack of standardized treatment guidelines in various species of birds is a major challenge to the veterinary profession. There is no agreed best choice of antifungal therapy to use and no standardized period of treatment and success criteria in the treatment [246]. The formation of evidence-based clinical guidelines using multi-center research would improve consistency and outcomes of treatment in a variety of avian populations.

## 5. Conclusions

*Candida* species are recognized as major opportunistic pathogenic agents in avian medicine, resulting in localized and systemic infections in domestic, companion, and wild birds worldwide. Despite *Candida* spp. being a normal part of the gastrointestinal microbiota of healthy birds, many *Candida* species, especially *C. albicans*, and a rising number of NACS are associated with invasive candidiasis in cases of compromised host defense. The opportunistic nature of avian *Candida* infection is indicated by their prevalence among poultry, psittacines, passerines, waterfowl, and wild birds.

Avian candidiasis pathogenesis is implicated with molecular virulence factors such as adhesin-mediated binding (ALS family, HWP1), yeast-to-hypha morphogenesis, tissue damage by Candidalysin, biofilm formation on mucosal and abiotic surfaces, and secreted hydrolytic enzymes (SAPs, phospholipases). Predisposing factors such as immunosuppression, malnutrition, long-term antibiotic treatment, poor husbandry, and crop stasis are the major factors that lead to the transition from commensalism to pathogenicity, especially in hand-reared psittacines and young poultry.

Diagnosis methods range from cytological analysis and fungal culture on selective media to more sophisticated methods such as PCR and MALDI-TOF MS, allowing accurate species identification and the detection of antifungal-resistance markers. The susceptibility data on antifungals reveal that nystatin and amphotericin B are still active against most avian isolates, with nystatin appearing as the first-line treatment of localized infection. Nevertheless, non-albicans *Candida* species are rising in azole resistance, thus requiring species-level identification to guide therapy.

The zoonotic potential of avian *Candida* reservoirs warrants attention. Wild birds, especially pigeons (*Columba livia*) may spread pathogenic yeasts to the environment and humans via aerosols of dried droppings and microbial colonization of feathers, feet, and beaks. Notably, molecular comparison of *C. dubliniensis* isolates from seabird excreta and human clinical samples has revealed that avian and human populations are largely genetically distinct, with cross-species transmission appearing to be rare and potentially occurring primarily from humans to birds. The risk of increasing antifungal resistance among domestic bird populations emerges as a threat because of the economic consequences of the poultry industry and the health impact on companion birds.

Prevention remains the cornerstone of disease control, emphasizing adequate sanitation, proper nutrition, and judicious antimicrobial use. Numerous knowledge gaps remain, such as a lack of avian-specific pharmacokinetic data, poor knowledge of species-specific virulence phenotypes, and the absence of point-of-care diagnostics. It is thus significant to implement meaningful treatment methods in mitigating the prevalence of resistance. Moreover, because of their widespread occurrence in routine ecological environments and known genetic plasticity, further molecular monitoring is justified to clarify whether the environmental or avian reservoirs of *Candida* spp. are the cause of the epidemiology of human candidiasis. By extension, improving mycological surveillance in One Health that considers veterinary and wildlife health would provide a more rigorous insight into the processes of potential interspecies transmissions.

## Figures and Tables

**Table 1:** Examples of avian species and their corresponding isolated *Candida* species.

Bird Species (Scientific Name)	Isolated *Candida* Species	References
Struthio camelus (ostrich)	No peer-reviewed *Candida* isolation located; ostrich fungal disease overviews exist (non-specific), and recent work profiles anaerobic gut fungi (not *Candida*)	[32,33]
Gyps fulvus (griffon vulture)	*Candida* spp. (oral mycoses; multiple yeast genera isolated from lesions)	[25]
Milvus milvus (red kite)	*C. lusitaniae* (first oral candidiasis report)	[34]
Nymphicus hollandicus (cockatiels)	*C. albicans*, *C. tropicalis*, *C. parapsilosis*, *C. krusei* isolates from crop/cloaca/oral cavity	[35]
Streptopelia capicola (ring-necked dove)	*C. glabrata* (GI disease case)	[23]
Branta canadensis (Canada goose)	No species-level *Candida* isolation specific to Canada goose located; waterfowl studies show *Calbicans* esophageal mycosis (not species-specific) and older reports of *Candida* in aquatic birds	[36]
Amazona spp. (parrots, various)	*C. albicans*, *C. glabrata*, *C. tropicalis*, *C. krusei* (from pet parrots; oral/cloacal isolates)	[37]
Gallus domesticus (domestic chicken)	*C. albicans* (oral cavity; confirmed molecularly)	[38]
Amazona auropalliata (yellow-naped Amazon)	*C. glabrata* (GI disease case)	[23]
Pionus senilis (white-crowned parrot	*C. krusei* (GI disease case)	[23]
Ara ararauna (blue-and-gold macaw)	*C. glabrata* (GI disease case)	[23]
Falco tinnunculus (common kestrel)	No *Candida* isolation located; microbiome studies exist (not *Candida*-focused isolation)	[39]
Columba livia (rock pigeon)	*Candida* spp. including *C. guilliermondii* (dominant), *C. albicans*; isolates from crop/cloaca/droppings	[7,40]

**Table 2:** Summary of antifungal susceptibility data from avian *Candida* isolates.

Reference	Avian Host	*Candida* spp.	Antifungal	MIC/ Susceptibility	Method
[228]	Eclectus parrot	*C. glabrata*	Nystatin	2 μg/mL (S)	Broth microdilution
			Fluconazole	32 μg/mL (SDD)	
[8]	Rock pigeon	*C. parapsilosis*	Fluconazole	0.25–0.5 μg/mL (S)	Broth microdilution (CLSI M27-A3)
			Itraconazole	≤0.125 μg/mL (S)	
			Amphotericin B	0.031–0.5 μg/mL (S)	
			Micafungin	≤0.03–0.125 μg/mL (S)	
		*C. tropicalis*	Fluconazole	≥64 μg/mL (R, 94.4%)	
			Itraconazole	≥64 μg/mL (R, 94.4%)	
		*C. krusei*	Fluconazole	≥8–64 μg/mL (R)	
[229]	Laying hens	*C. albicans* (*n* = 84)	FLC, ITZ, VOR, POS	High MIC (MDR 26%)	Broth microdilution (CLSI)
		*C. catenulata* (*n* = 17)	ITZ, POS, MCF, ANI	R	
			Amphotericin B	S	
[230]	Poultry environment (Nigeria)	*C. parapsilosis*	Nystatin	S (19–25 mm)	Disk diffusion
			Fluconazole	S (19–25 mm)	
			Voriconazole	S (19–25 mm)	
[37]	Pet parrots	*Candida* spp. (*n* = 12)	Ketoconazole	R (25%)	Disk diffusion
			Fluconazole	R (50%)	
			Miconazole	R (12.5%)	
			Clotrimazole	R (12.5%)	
[38]	Domestic chicken	*C. albicans*	AmB, FLC, 5-FC, VOR, CAS, MCF	S (all isolates)	Vitek 2 automated
[4]	Multiple species *	*C. albicans* (*n* = 28)	Nystatin	S (78.5%)	Disk diffusion (CLSI)
			Amphotericin B	S (78.5%)	
			Fluconazole	R (78.5%)	
			Ketoconazole	R (75%)	
			Itraconazole	R (53.5%)	
		*NACS* (*n* = 21)	Nystatin	S (71.4%)	
			Amphotericin B	S (61.9%)	
			Fluconazole	R (52.3%)	
			Itraconazole	R (66.6%)	

Abbreviations: AmB, amphotericin B; FLC, fluconazole; ITZ, itraconazole; VOR, voriconazole; POS, posaconazole; MCF, micafungin; ANI, anidulafungin; CAS, caspofungin; 5-FC, flucytosine; S, susceptible; SDD, susceptible dose-dependent; R, resistant; MDR, multidrug resistant; NACS, non-albicans *Candida* species. * Multiple species include turkey, chicken, partridge, mallard, pigeon, cockatiel, lovebird, grey parrot, common myna, white-eared bulbul, and zebra finch.

## Data Availability

No new data were created or analyzed in this study. Data sharing is not applicable to this article.
